# Elucidating tissue and subcellular specificity of the entire SUMO network reveals how stress responses are fine-tuned in a eukaryote

**DOI:** 10.1126/sciadv.adw9153

**Published:** 2025-08-27

**Authors:** Jason Banda, Shraboni Ghosh, Dipan Roy, Kishor D. Ingole, Lisa Clark, Eshan Sharma, Sumesh Kakkunath, Kawinnat Sue-Ob, Rahul Bhosale, Leah Band, Srayan Ghosh, Darren Wells, Jonathan Atkinson, Nicholas J. Provart, Malcolm J. Bennett, Kathryn S. Lilley, Andrew Jones, Miguel De Lucas, Anthony Bishopp, Ari Sadanandom

**Affiliations:** ^1^ Plant & Crop Sciences, School of Biosciences, University of Nottingham, Loughborough LE12 5RD, UK.; ^2^ Department of Biosciences, Durham University, Durham DH1 3LE, UK.; ^3^ Department of Biochemistry, University of Cambridge, Cambridge CB2 1RX, UK.; ^4^ Institute of Systems, Molecular and Integrative Biology, Liverpool L69 3BX, UK.; ^5^ Department of Biology, University of York, York YO10 5DD, UK.; ^6^ Centre for Mathematical Medicine and Biology, School of Mathematical Sciences, University of Nottingham, Nottingham NG7 2RD, UK.; ^7^ Department of Cell & Systems Biology/Centre for the Analysis of Genome Evolution and Function, University of Toronto, Toronto, Ontario M5S 3B2, Canada.

## Abstract

SUMOylation is essential in plant and animal cells, but it remains unknown how small ubiquitin-like modifier (SUMO) components act in concert to modify specific targets in response to environmental stresses. In this study, we characterize every SUMO component in the *Arabidopsis* root to create a complete SUMO Cell Atlas in eukaryotes. This unique resource reveals wide spatial variation, where SUMO proteins and proteases have subfunctionalized in both their expression and subcellular localization. During stress, SUMO conjugation is mainly driven by tissue-specific regulation of the SUMO E2-conjugating enzyme. Stress-specific modulation of the SUMO pathway reveals unique combinations of proteases being targeted for regulation in distinct root tissues by salt, osmotic, and biotic signals. Our SUMO Cell Atlas resources reveal how this posttranslational modification (PTM) influences cellular- and tissue-scale adaptations during root development and stress responses. To our knowledge, we provide the first comprehensive study elucidating how multiple stress inputs can regulate an entire PTM system.

## Teaser

A spatial map of an entire protein modification system reveals tissue and subcellular diversification of SUMO components.

## INTRODUCTION

Posttranslational modifications (PTMs) are crucial in orchestrating biological complexity and modulating nearly every biological process. This is particularly evident in multicellular organisms where development requires precise coordination of cell fates (*1*). There are >100 different PTMs, including ubiquitination and phosphorylation (*1*–*3*). Unraveling the mechanisms regulating these PTMs can be challenging due to the very large number of components involved. However, compared with the many hundreds of proteins involved in ubiquitination, experimentally characterizing the 30 to 40 components required for the SUMO (small ubiquitin-like modifier) pathway is far more tractable. SUMOylation is a key mechanism for rapid control of protein degradation, activity, localization, and binding partners (*4*, *5*), regulating an array of complex biological processes, from stress perception to chromatin modification and transcription (*6*–*8*). Given its importance in cell function and its tractable genetics, SUMO allows us to delve deeper into how multicellular organisms use PTMs to integrate stress signals by modifying key substrates to coordinate downstream effects on adaptive responses.

SUMO is ~11 kDa in molecular weight, slightly larger than ubiquitin, and is found in all eukaryotes. The SUMO modifier protein has a tertiary structure consisting of the β-grasp fold orientation structure (*9*), which is also common to other protein conjugation systems such as ubiquitin and neuronal-precursor-cell-expressed developmentally downregulated protein-8 (NEDD8). SUMO genes are translated as premature peptides with C-terminal extensions to form a diglycine motif. The C-terminal end is processed to expose a diglycine terminus, which is then conjugated to lysine residues in target substrates through a cascading collection of enzymes sequentially named E1, E2, E3, and E4. SUMO is covalently linked to its substrate as single or multiple monomers or as polymers composed of different SUMO peptides catalyzed by the E4 enzyme (*10*). The SUMO E2 enzyme can also facilitate SUMO conjugation directly to its substrate protein or to the SUMO E3 ligase for conjugation onto substrates. The ubiquitin-like protease (ULP) class of SUMO proteases can process premature SUMO and remove SUMO from substrates, while deSUMOylating isopeptidases (DeSIs) exclusively remove SUMO from targets. Together, these proteases regulate the pool of free and conjugated SUMO within cells (fig. S1).

Families of all major components of the SUMO cycle, except ubiquitin specific peptidase like 1 (USPL1) proteases, are present in multiple eukaryotic kingdoms, suggesting similar functions. However, there are some noticeable differences between the animal and plant SUMO systems. Animal SUMO systems have an increased number of E3 ligases—eight genes—whereas in plants, only two have been confirmed to date (*11*, *12*). Conversely, plants have a more extensive number of SUMO peptides and proteases than animals (*9*). The expansion of SUMO ligase genes in animals and protease genes in plants may provide insights into how a single PTM system diverged to suit each kingdom’s different developmental and adaptive needs.

Despite SUMO modification enzymes being well studied in both plant and animal kingdoms, it is unknown how SUMO components act in concert to modify their targets in response to changing environments. Furthermore, despite their critical importance in numerous cellular processes, our understanding of the rules governing SUMO target specificity remains rudimentary. We use the root of the plant *Arabidopsis thaliana* as a model to address this. We report the spatial expression and protein localization of every SUMO system component to create a complete SUMO Cell Atlas (SCA) and reveal how SUMO influences cellular- and tissue-scale adaptations during plant development and stress responses. In doing so, we provide a comprehensive network elucidating how multiple inputs can regulate a single PTM.

## RESULTS

### Simultaneous visualization of SUMO gene expression and protein localization

To understand the role of SUMO in regulating cellular states, fates, and molecular processes, it is crucial to map the spatiotemporal distribution of the SUMO-machinery components (fig. S1) within a model organ. Given its simple and robust cellular organization, the *Arabidopsis* primary root is an ideal organ for developing an SCA. The *Arabidopsis* primary root is derived from the root apical meristem, which is made up of proximal and distal stem cells separated by an organizing center (the quiescent center). The proximal portion of the root comprises a series of distinct cell types (epidermis, cortex, endodermis, and pericycle) arranged in concentric layers around a central vascular cylinder (comprising phloem, xylem, and procambial cells) (*13*). As new cells divide in the meristematic stem cell niche, mature cells are displaced away from this zone. These displaced cells then undergo a period of expansion before differentiating into their final cell fates. The timing of each phase of root cell development (division, elongation, and differentiation) is tightly controlled by genetic and environmental factors, resulting in three distinct zones within the root tip: meristem, elongation zone (EZ), and differentiation zones (*13*).

We analyzed published single-cell RNA sequencing (scRNA-seq) data and observed differential expression for all SUMO components in different cell types (fig. S2). For example, *SUMO3* is expressed in epidermal cells but not in the vasculature and quiescent center cells. *SUMO3* expression correlated with the expression of SUMO proteases *SPF1*, *OTS2*, and *DeSI3B*, with high expression only in the meristem zone, indicating a potential functional relationship between these components. To validate the observed coexpression-based functional association between the SUMO components across different cell types, we needed to simultaneously analyze gene expression, protein abundance, and subcellular localization of each component.

### Atlas reveals subfunctionalization of SUMO peptides and DeSI proteases

**Fig. 1.**
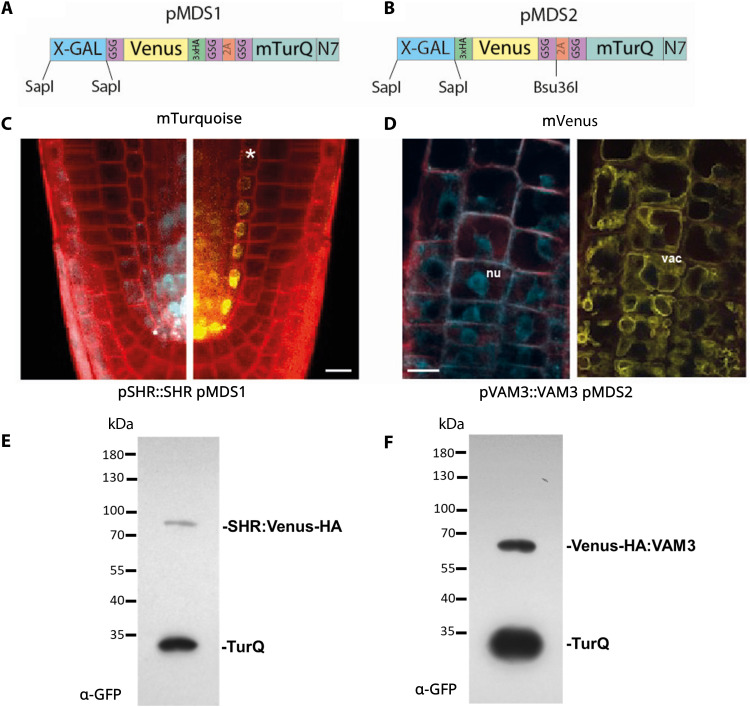
Description and validation of pMDS plasmid system for dual analysis of transcription and translation in plants. (**A**) Organization of pMDS1 vector showing reporters for transcription [mTurquoise (mTurQ)], translation (C-terminal mVenus), and a 2A self-cleaving peptide. (**B**) Organization of pMDS2 vector showing reporters for transcription (mTurQ), translation (N-terminal mVenus), and a 2A self-cleaving peptide. (**C**) Confocal image of pMDS1_*SHR*pro:*SHR:mVenus:mTurQ* showing gene expression (mTurQ) in the stele region and protein (mVenus) translocating to endodermis in the root meristem. (**D**) Confocal image of pMDS2_*VAM3*pro:mVenus:*VAM3:mTurQ* showing subcellular expression (mTurQ) in the nucleus and protein (mVenus) moving to the vacuole in the root epidermis. Red channel shows mCherry expression. nu, nucleus; vac, vacuole; *, endodermis of root meristem. Scale bar, 10 μM. (**E**) Immunoblot of pSHR::SHR pMDS1 showing efficient ribosomal skipping induced by the 2A peptides. Bands corresponding to mTurquoise are labeled as mTurQ. (**F**) Immunoblot of pVAM3::VAM3 pMDS2 showing efficient ribosomal skipping induced by the 2A peptides. Bands corresponding to mTurquoise are labeled as mTurQ.

To generate an expression atlas for every *Arabidopsis* SUMO component, we synthesized transcriptional and translational fluorescent fusions for all 32 SUMO cycle genes using an innovative reporter design. This dual transcriptional and translational reporter contained the endogenous promoter region for the respective SUMO cycle gene, followed by the genomic sequence fused to a hemagglutinin (HA)–tagged mVenus-3xHA-2A-mTurquoise-N7 dual reporter (Fig. 1A). The 2A peptide induces ribosomal skipping during translation, creating a SUMO-mVenus-HA translational reporter and a cotranscribed, nuclear-localized mTurquoise reporter, both driven by the same promoter (*14*–*16*). The translational reporter will denote the spatial domain, at both (sub)cellular and tissue scales, in which the protein is located. The second peptide, mTurquoise-N7, defines the spatial domain where transcription/translation of the gene occurs. Any differences in the spatial location of the two fluorescent signals will reveal whether SUMO proteins exhibit cell-to-cell movement or whose abundance is regulated via degradation.

To validate the functionality of our polycistronic reporter approach, we analyzed the transcriptional and translational behavior of two well-characterized genes: SHORT-ROOT (SHR) and VACUOLAR MORPHOLOGY 3 (VAM3). SHR is a transcription factor that regulates root radial patterning via its non–cell-autonomous behavior (*17*). The SHR gene is initially transcribed in the vascular cells of the root apical meristem, but its protein moves into adjacent endodermal and cortex initials (*17*). The *SHR* promoter and genomic coding region were cloned into the vector (termed pMDS1; Fig. 1A) to create a C-terminal reporter fusion. Transgenic plants exhibited an mTurquoise-N7 signal in the stele (denoting the SHR transcription domain), while the mVenus signal (denoting SHR protein distribution) was expanded to include endodermal and cortex initials (Fig. 1, A and C), demonstrating SHR protein movement and validating our dual-reporter approach. Furthermore, efficient 2A peptide skipping of the construct was verified using VAM3, a Q-SNARE receptor that locates to vacuolar and prevacuolar compartments cloned into a vector variant termed pMDS2. In transgenic roots, VAM3 gene expression (visualized with mTurquoise-N7) was present in the nucleus of epidermal cells, while the VAM3 protein was only visible in the vacuole of these cells (Fig. 1, B and D). In addition, Western blot analysis of protein extracts from VAM3 pMDS2 transgenic lines demonstrated efficient ribosome skipping conferred by the 2A peptide signal (Fig. 1, E and F), as no hybrid peptide at higher molecular weight was detected. These results indicate that the pMDS vector system works efficiently and allows us to simultaneously visualize expression and protein localization. This vector system therefore enabled us to ascertain whether rapid changes in gene expression or protein levels of SUMO components are due to induction and/or degradation.

**Fig. 2.**
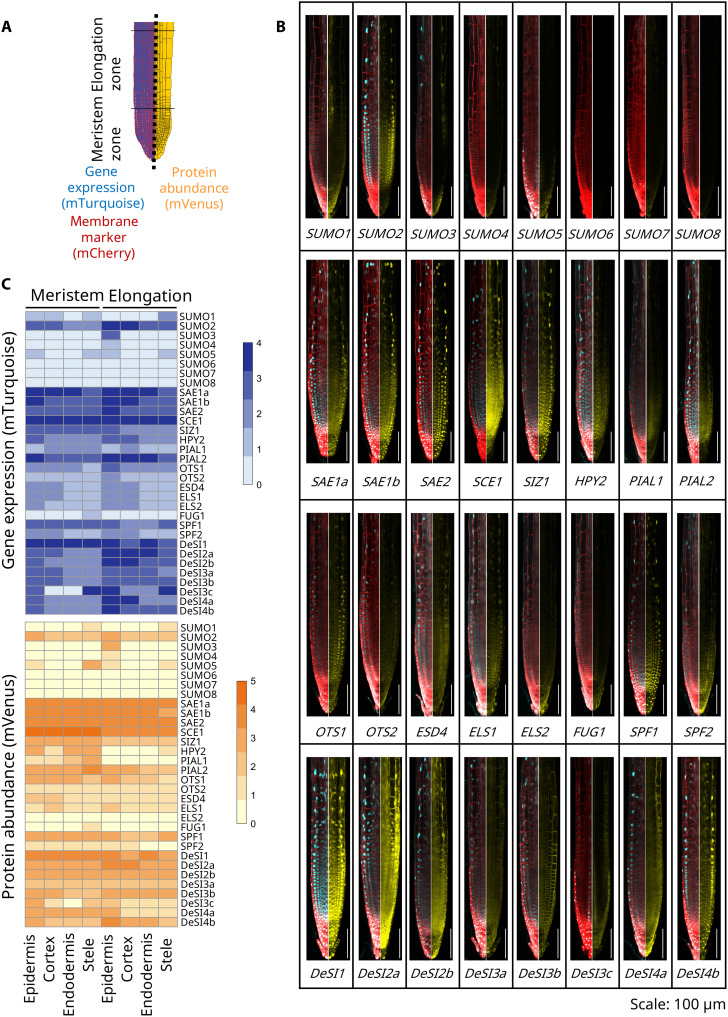
SUMO components exhibit distinct domains of expression and protein accumulation in *Arabidopsis* root tips. (**A**) Schematic representation of the root tip zones and tissues measured in the *Arabidopsis* root. (**B**) Confocal images displaying gene expression (cyan) and protein localization (yellow) of all 32 SUMO components in the root tip of *A. thaliana*. The cell outline is shown in red by a genetic membrane marker. Scale bars, 100 μm. (**C**) Heatmap representing the semiquantitative level of gene expression and protein abundance of all SUMO components in the varying root zones and tissues (*n* = 5). Zero indicated no fluorescence observed, while level 4 or 5 defines a high level of fluorescence. For (B), image settings have been optimized for individual lines to allow comparison of expression patterns; for (C), image settings are identical between lines to allow quantitative comparisons between components.

To create a SUMO Cell Atlas for a plant organ, we characterized each component’s expression and subcellular localization in the key *Arabidopsis* root cell types (i.e., epidermis, cortex, endodermis, and vasculature) and zones (i.e., meristem and elongation; Fig. 2, A and B). We quantified each SUMO component’s expression at both transcription and translation (using mTurquoise) and protein (using mVenus) levels in each root cell type and zone and then collated these values into five categories of expression (low to high; Fig. 2C). This approach allows us to define levels of each SUMO component per tissue and zone. To validate that the genomic fragments of the SUMO components cloned into the pMDS vectors faithfully replicated endogenous gene expression, we performed quantitative reverse transcription polymerase chain reaction (qRT-PCR) to compare transgene and endogenous SUMO gene expression. Two independent transgenic lines for each SUMO component exhibiting similar gene expression patterns to the corresponding endogenous gene were taken forward for further analysis (fig. S3). Furthermore, efficient 2A peptide skipping of each of the SUMO component constructs was verified using immunoblot analysis (figs. S4 to S7). Last, to validate that SUMO components tagged with mVenus remain functional, we successfully complemented mutants in SUMO components with known phenotypes (figs. S8 and S9).

The SUMO Cell Atlas revealed several surprising insights. Earlier work assumed that *SUMO1* and *SUMO2* were ubiquitously expressed within root tissues (*18*), but using our reporter lines, we observed discrete expression patterns for these two modifiers. *SUMO2* was ubiquitously expressed in all root cell types and zones measured. In notable contrast, SUMO1 exhibited a much more restricted pattern, with high levels of expression in a subset of vasculature cells in the EZ, which we confirmed as phloem companion cells after analyzing root cross sections (fig. S10). scRNA-seq datasets (fig. S2) supported both patterns. These differences in transcriptional regulation of SUMO1 and SUMO2 at the spatial level provide a basis for subfunctionalization, where these modifiers mediate responses to different stimuli. Discrete expression patterns were also observed for other SUMO peptides in our reporter lines. *SUMO3* was detected in epidermal cells within the EZ. *SUMO4* was expressed at very low levels in the late EZ, and *SUMO5* demonstrated very low expression in vasculature and epidermis. The remaining *SUMO6*, *SUMO7*, and *SUMO8* were not detected in the root tip yet could be involved in older root tissue or in shoot organs. This wide variation in the spatial expression of SUMO genes could enable cell-type–specific conjugation of targets.

E1 to E4 enzymes form the core SUMO conjugation machinery. The genes encoding these key components exhibited the highest levels of expression and protein abundance in the SUMO cycle, especially SAE1a, SAE1b, SAE2, and SCE1. The E3 HIGH PLOIDY2 (HPY2) and E4 ligase PROTEIN INHIBITOR OF ACTIVATED STAT LIKE1 (PIAL1) had the lowest expression, and the E3 ligase SAP AND MIZ1 (SIZ1) was intermediate in expression in all root tissues and zones analyzed. HPY2 is expressed in both root zones, but the protein is only visible in the meristematic zone. This observation is consistent with the reported role for HPY2 as a regulator of cell proliferation in the apical meristem where it functions as a repressor of the endocycle (Fig. 2B) (*19*, *20*). The SUMO chain–forming E4 ligase PIAL1 is expressed at low levels in multiple root cell types, but its protein is only found in early meristematic tissue. A number of the E1 to E4 enzymes are localized in both the nucleus and cytoplasm (figs. S11 and S12). This suggests that SUMOylation of targets could occur outside the nucleus, explaining the SUMOylation of plasma membrane targets such as Flagellin Sensitive2 (FLS2) (*21*).

ULP proteases perform a dual function: SUMO maturation through cleaving the preprotein to release the peptide and SUMO removal from its target substrates. In general, this group of proteases displayed low expression levels and protein abundance. Most ULPs are expressed in all root cell types; however, *FOURTH ULP GENE CLASS 1 (FUG1)* is predominantly observed in pericycle cells (a layer of cells surrounding the vascular tissues that gives rise to lateral roots), where FUG1 might play a role in epigenetic gene silencing (*22*). SUMO maturation is considered to occur only in the nucleus; however, OVERLY TOLERANT TO SALT 2 (OTS2) and ESD4-LIKE SUMO PROTEASE 1 (ELS1) proteases are localized to the nuclear envelope (figs. S11 and S12). Previous work demonstrated binding of human SUMO specific proteases (SENPs) to nuclear pore complexes in the nuclear envelope, where the proteases are involved in localization and transport kinetics (*23*). Hence, OTS2 and ELS1 may perform similar roles in plants. ELS1 and EARLY IN SHORT DAYS4 (ESD4) are weakly localized in the cytoplasm. They may fulfill a dual role between peptidase activity in the nucleus and isopeptidase activity in the cytoplasm.

In contrast to the ULP class, DeSI proteases contain an isopeptidase domain that cleaves SUMO off substrates but does not function during SUMO maturation. This distinct family of DeSI proteases is more highly expressed and abundant than its ULP peptidase counterpart. DeSIs exhibit expression in all root tissues and zones with the exception of *DeSI3c*, which is limited to the lateral root cap and vasculature, with weak expression in the epidermis. DeSI showed a wide variety of subcellular locations (figs. S11 and S12). DeSI1 and DeSI4b were strongly localized to the nucleus with weak cytosolic abundance. DeSI2a and DeSI2b displayed similar protein levels in both the nucleus and cytosol. DeSI3a, DeSI3b, and DeSI3c are all localized to the plasma membrane, with DeSI3a and DeSI3c also containing a speckled signal in the cytosol, which might indicate specific target proteins (fig. S11). The exception is DeSI4a, which is localized to a discrete cellular compartment demonstrated by the speckled signal (fig. S11). Little is known about the molecular targets of these DeSIs, but our SUMO Cell Atlas reveals a strong subcellular variation, indicating that individual DeSIs may target a discrete set of targets in different cellular components. Our SUMO system-wide approach raises the possibility of a much more complex regulatory landscape for SUMO modification than previously known.

### Probing stress regulation of the SUMO Cell Atlas

The 32 dual-reporter lines within our Cell Atlas resource were next used to establish how the SUMO system modulates the SUMOylated proteome upon different environmental cues. We studied three model abiotic and biotic stresses: salt, osmotic and bacterial elicitor, and flagellin. For salt, osmotic, and flagellin treatment, seedlings were exposed to 150 mM NaCl, 300 mM mannitol, or 1 μM flagellin.

We first verified that SUMO systems respond after 3 hours of exposure to model stimuli. Immunoblot analysis confirmed SUMO conjugate accumulation 3 hours posttreatment (fig. S13). Next, we quantified changes in SUMO component gene expression 3 hours after treatment using bulk RNA-seq on whole roots (fig. S14). The salt and mannitol treatments have similar numbers of differentially expressed genes (DEGs; fig. S14A). Flagellin treatment, however, only triggered relatively mild transcriptomic changes. In the salt and mannitol treatment, Gene Ontology (GO) analysis revealed that up-regulated genes were enriched in osmotic homeostasis signaling pathways (response to salt, response to water deprivation, etc.) (fig. S14B). Flagellin treatment up-regulated known defense response genes but down-regulated glucosinolate and carbohydrate metabolism.

**Fig. 3.**
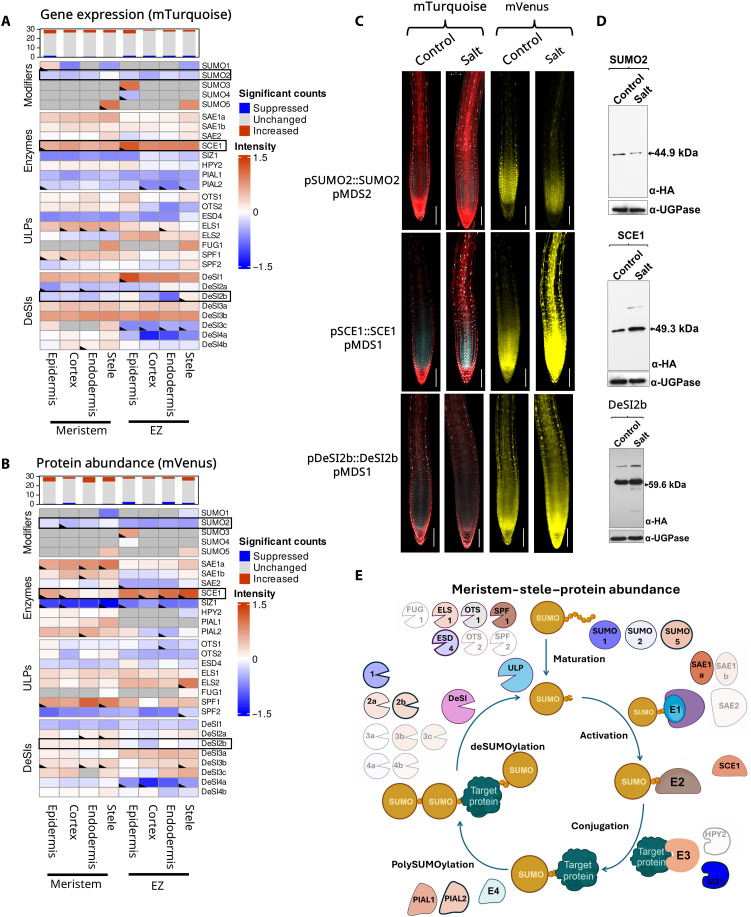
Salt stress modulates the SUMO system in a tissue-specific manner. (**A**) Heatmap representing the log_2_ fold changes in gene expression within the SUMO components 3 hours after 150 mM salt stress in several root tissues and zones (*n* = 4). The top bar graph displays the number of SUMO genes per tissue type in a specific zone that change in a statistically significant manner (*P* ≤ 0.05). Dark gray boxes indicate combinations that were not analyzed, as no expression or protein was observed in this tissue. (**B**) Similar heatmap as in (A) representing the log_2_ fold changes in protein abundance within the SUMO components 3 hours after 150 mM salt stress in several root tissues and zones. (**C**) Confocal microscopy images showing changes in expression (mTurquoise: cyan) and protein abundance (mVenus: yellow) of three major changers during salt stress. Scale bars, 100 μm. (**D**) Western blot of three major SUMO changers during salt stress, confirming the trend as seen in image analysis. UTP--glucose-1-phosphate uridylyltransferase (UGPase) was used as a loading control. (**E**) Schematic representation of the SUMO cycle and its components. The cycle represents the changes in protein abundance in the meristematic stele. Components with modest changes (fold change < 0.5) are shown as transparent, while those with the largest changes (log change > 0.5) are shown in solid color based on the scale of the heatmap in (B). Components with statistically significant changes are also outlined.

**Fig. 4.**
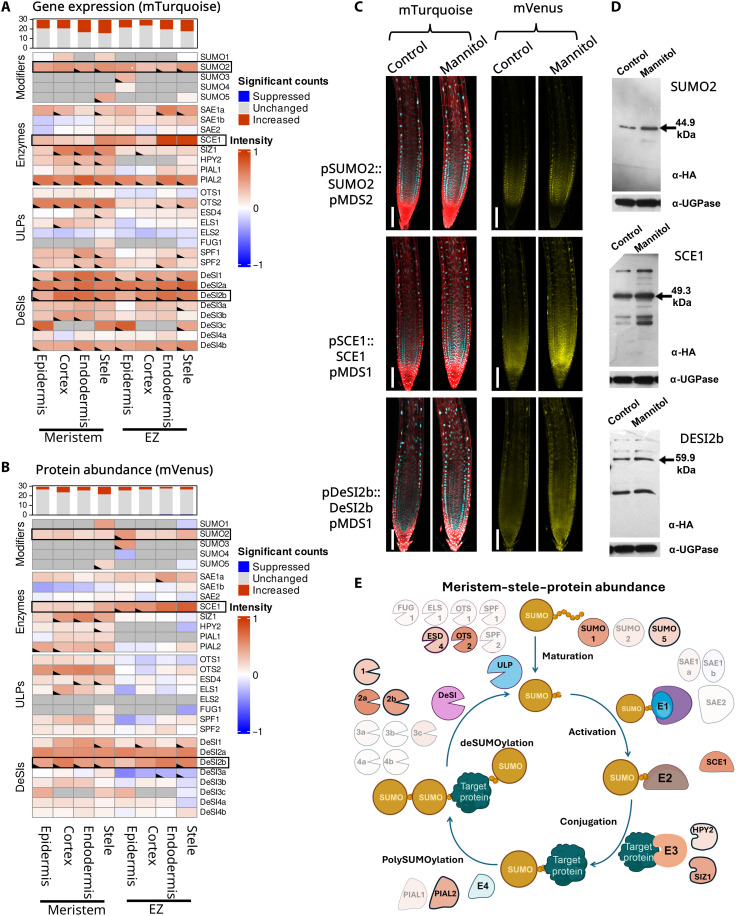
Osmotic stress regulates a distinct set of genes compared to salt stress. (**A**) Heatmap representing the log_2_ fold changes in gene expression within the SUMO components 3 hours after 300 mM mannitol stress in several root tissues and zones (*n* = 4). The top bar graph displays the number of SUMO genes per tissue type in a specific zone that change in a statistically significant manner (*P* ≤ 0.05). Dark gray boxes indicate combinations that were not analyzed, as no expression or protein was observed in this tissue. Boxes with a marked corner indicate significant changes. (**B**) Similar heatmap as in (A) representing the log_2_ fold changes in protein abundance within the SUMO components 3 hours after 300 mM mannitol stress in several root tissues and zones. (**C**) Confocal microscopy images showing changes in expression (mTurquoise: cyan) and protein abundance (mVenus: yellow) of three major changers during salt stress. Scale bars, 100 μm. (**D**) Western blot of three major SUMO changers during salt stress, confirming the trend as seen in image analysis. UGPase was used as a loading control. (**E**) Schematic representation of the SUMO cycle and its components. The cycle represents the changes in protein abundance in the meristematic stele. Components with modest changes (fold change < 0.5) are shown as transparent, while those with the largest changes (log change > 0.5) are shown in solid color based on the scale of the heatmap in (B). Components with statistically significant changes are also outlined.

**Fig. 5.**
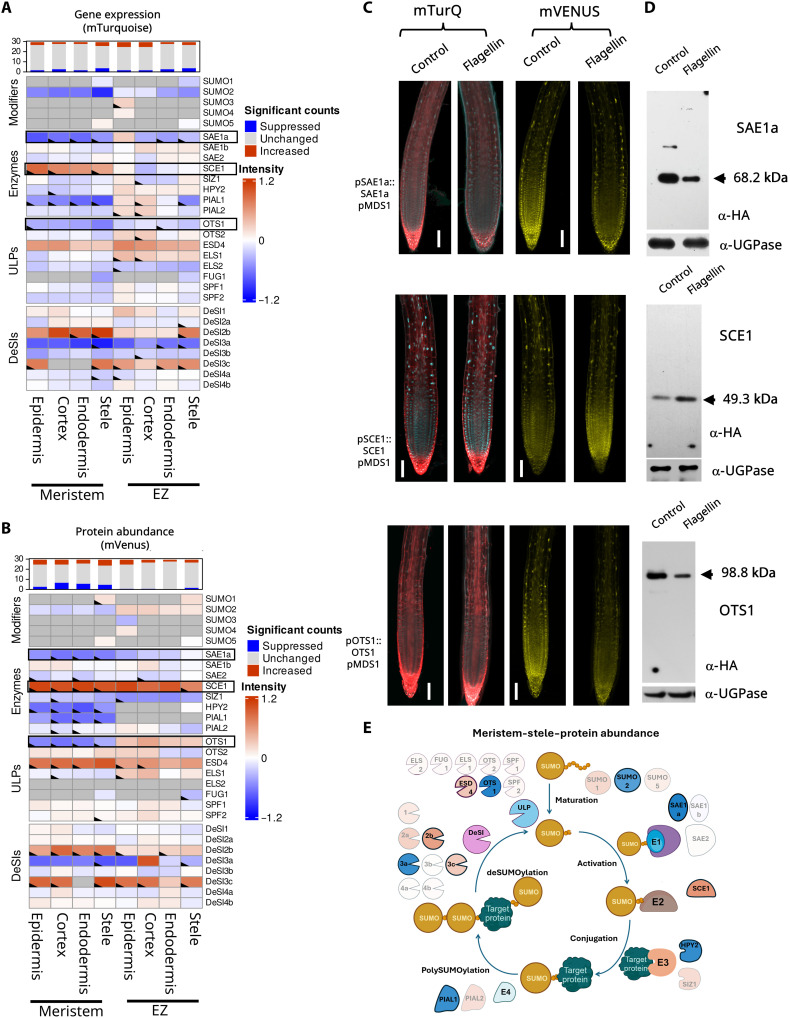
Biotic stress targets a distinct subset of SUMO system genes compared to abiotic stress. (**A**) Heatmap representing the log_2_ fold changes in gene expression within the SUMO components 3 hours after 1 μM flagellin treatment in several root tissues and zones (*n* = 4). The top bar graph displays the number of SUMO genes per tissue type in a specific zone that change in a statistically significant manner (*P* ≤ 0.05). Dark gray boxes indicate combinations that were not analyzed, as no expression or protein was observed in this tissue. Boxes with a marked corner indicate significant changes. (**B**) Similar heatmap as in (A) representing the log_2_ fold changes in protein abundance within the SUMO components 3 hours after 1 μM flagellin treatment in several root tissues and zones. (**C**) Confocal microscopy images showing changes in expression (mTurquoise: cyan) and protein abundance (mVenus: yellow) of three major changers during 1 μM flagellin treatment. Scale bars, 100 μm. (**D**) Western blot of three major SUMO changers during 1 μM flagellin treatment, confirming the trend as seen in image analysis. UGPase was used as a loading control. (**E**) Schematic representation of the SUMO cycle and its components. The cycle represents the changes in protein abundance in the meristematic stele. Components with modest changes (fold change < 0.5) are shown as transparent, while those with the largest changes (log change > 0.5) are shown in solid color based on the scale of the heatmap in (B). Components with statistically significant changes are also outlined.

The same treatments at a 3-hour time point were used to visualize the response of the 32 SUMO component reporters. Seedlings were scanned on the confocal microscope to visualize both mTurquoise and mVenus patterns. The abundance of every SUMO component was quantified in multiple cell types, including epidermis, cortex, endodermis, and stele, and within two root zones, meristem and EZ. The changes induced by each stress within these cell types were visualized as expression (Figs. 3 to 5A) and protein abundance (Figs. 3 to 5B) heatmaps to reveal common and contrasting spatial and quantitative regulatory effects on each SUMO component.

### SUMO in epidermal cells is most responsive during salt stress

Salt stress profoundly affects root growth and development (*24*, *25*). It is well documented that salt stress induces SUMO conjugation to substrate proteins in roots (fig. S13) (*26*, *27*). However, it remains unclear how the SUMO system responds to salt stress. SUMO Cell Atlas resources revealed that after 3 hours of salt stress, SUMO1 and SUMO2 were down-regulated (both gene expression and protein abundance), while SUMO3 and SUMO5 showed slight up-regulation (Fig. 3). The SUMO1 protein was strongly inhibited in the stele of the meristem, while SUMO2 was reduced across all cell types in the EZ (Fig. 3, A and B). This spatial specificity in down-regulation suggests specific developmental roles for these two highly similar SUMO isoforms in salt response. The observation that SUMO3 and SUMO5 protein levels increase following both osmotic and salt stress suggests that these two SUMOs might be involved in specific stress response pathways other than targets of SUMO1 and SUMO2.

Among the SUMO conjugation enzymes, the E2 enzyme, SCE1, showed the greatest induction after salt stress (Fig. 3, C and D). We observed significantly increased levels of SCE1 protein in the EZ compared to the meristem, with the highest expression in the epidermis and protein abundance in the stele. The epidermis is where salt is first perceived in the roots, so SCE1 induction in this tissue suggests a direct role for salt perception by the SUMO system. The E3 ligase SIZ1 was strongly down-regulated across all cell types and zones, while HPY2 levels remain stable. These results suggest that during salt stress, SUMOylation relies more on increased levels of E2. *PIAL1* and *PIAL2* showed decreased expression but increased protein abundance. These proteins are likely stabilized during salt stress, leading to increased poly-SUMO formation.

Among the SUMO proteases, OTS1 and OTS2 showed a clear reduction in protein levels consistent with earlier studies on this subset of proteases, indicating that they are degraded after salt stress (*26*). ELS1 and SUMO PROTEASE RELATED TO FERTILITY1 (SPF1) showed increased gene expression and protein abundance. As *SPF1* gene expression is not strongly induced, this increase in protein abundance is likely regulated through increased protein stability under these conditions. SPF1 was strongly induced specifically in the endodermis and stele tissues. This implies a possible role for this ULP protease in regulating salt responses in inner root tissues. Among the DeSI-type proteases, the highly expressed DeSI1 transcripts were up-regulated in both meristem and EZ, primarily in the epidermal layer, although their protein levels decreased under salt stress. Likewise, DeSI4a protein also showed a strong reduction in cortex and endodermis. This suggests that ULP and DeSI proteases play specialized roles in different cell types under salt stress to shape their SUMOylated proteomes (Fig. 3E).

### Osmotic stress modulates SUMOylation primarily in inner root tissues

Osmotic stress treatment up-regulated both SUMO peptide and SUMO ligase components (Fig. 4, A and B), consistent with previous work demonstrating increased SUMOylation in response to abiotic stresses (*28*–*30*). Both salt and osmotic stresses induce expression of *SAE1a*, *SCE1*, *SPF1*, *and DeSI1*. The increase in SCE1 protein abundance stands out in both datasets. During osmotic stress, SUMO2 modifier abundance is increased (Fig. 4, C and D). This trend could be the basis for the increase in SUMO conjugation seen upon osmotic stress treatment (fig. S13). In *SUMO2* and *SCE1*, the greatest changes in expression occur in inner tissues (endodermis and stele) of the EZ, suggesting a local function responding to osmotic changes within these root tissues.

Strong increases in the expression and protein abundance of DeSI proteases 2a and 2b were also observed (Fig. 4, A and B). Whereas DeSI2a displays an increase in all tissues and zones, DeSI2b increases mostly in endodermis and stele cell types, similar to SUMO2 and SCE1. This result reveals potential roles for SUMO2 and SCE1 during osmotic stress by up-regulating the SUMOylation of a wide group of targets, while DeSI2a and DeSI2b deSUMOylate specific targets to modulate stress response pathways.

Transcript and protein heatmaps reveal interesting zone-specific responses to osmotic stress for selected SUMO components (Fig. 4, A and B). For example, protein abundance of many ULP and DeSI proteases in the meristem is stable or increased after 3 hours of osmotic stress. However, in the EZ, a drop is visible for proteases such as OTS1, FUG1, SPF1, and DeSI3a (Fig. 4B). In the case of OTS1 and FUG1, this can be directly ascribed to a decrease in gene expression (Fig. 4A). This contrasting behavior suggests active breakdown of the protein in the EZ specifically during osmotic stress. In both SPF1 and OTS1, this trend is clearest in epidermal cells, which would perceive the stress first. The notable differences between root meristem and EZ highlight the importance of these two proteases in rapid osmotic stress responses.

Following osmotic stress treatment, most SUMO component expression changes are found in inner root tissues. Around 50% of the SUMO-machinery genes showed significantly increased expression levels in the endodermis and stele of the root meristem versus ~40% in the EZ. Significant changes in protein abundance were rarer, with the highest level of change in the meristem stele cells where 26% (7 of 26) of proteins increased versus two proteins increased (DeSI1 and DeSI2b) and one (DeSI3a) was suppressed in the EZ. The meristem, therefore, appears to be the most reactive tissue when focusing on SUMO components. Focusing specifically on which proteins change within the stele, we observe changes in almost all E3 and E4 ligases (HPY2, SIZ1, and PIAL2) and changes in SUMO1, SUMO5, and SCE1. These changes are likely to cause an increased level of SUMO conjugation within stele cells. In contrast, levels of many ULP and DeSI proteases remain unchanged (Fig. 4E). Only DeSI1, DeSI2b, and ESD4 show significant increases. The elevation of SUMOylation versus deSUMOylation components likely explains the increased levels of SUMOylation observed after dehydration and osmotic stress. However, deSUMOylation of specific targets mediated by the increased abundance of DeSI components could play a vital role in activating specific stress pathways.

### Biotic stress predominantly affects SUMO components in the meristem

In parallel with our abiotic stress treatments, we observed that the biotic stress elicitor flagellin 22 (flg22) triggers dynamic changes of specific SUMO components primarily in the meristematic zone in a cell-type–specific manner (Fig. 5, A and B). Unlike abiotic stress treatments, changes in SUMO cycle machinery after flg22 application involved a smaller set of classes of components. The expression and protein abundance levels of all the SUMO modifiers remain unchanged, except for SUMO2, whose expression and protein abundance levels decreased in the meristematic zone (Fig. 5, C and D). Flagellin treatment decreased both transcription and protein abundance levels of SAE1a, particularly in the meristematic zone. In contrast, the expression and protein abundance levels of SAE1b and SAE2 largely remained unchanged. However, we observed an increase in the levels of the SUMO E2-conjugating enzyme, SCE1, in the cell in the meristem more prominently than the EZ (Fig. 5, A and B). Moreover, the protein abundance levels of the E3 ligase, HPY2, and the E4 ligase PIAL1 showed a slight but significant reduction in the meristematic zone.

Among the ULP proteases, there was a significant decrease in the protein abundance of OTS1 particularly in the meristematic zone, while ESD4 was up-regulated both at the transcriptional and translational levels (Fig. 5E). Among the components constituting the DeSI SUMO proteases, there was strong up-regulation at the transcriptional and translational levels for DeSI2b across all the examined tissue types in the meristematic zone. DeSI3c also constituted a candidate DeSI protease component that exhibited strong up-regulation, particularly at the protein abundance level upon flagellin treatment across all examined tissue types in the meristematic and EZ except the endodermis. Consistent with previous reports (*21*), flagellin treatment caused a strong decrease in the protein levels of DeSI3a. The response status of DeSI3a upon flagellin treatment served as an internal positive reference control for our study, confirming the efficiency of our experimental setup to elicit a biochemical and immune response in treated plants.

To explore expression patterns between the different SUMO components, we made gene and protein expression clusters based on imaging data on a tissue-specific level. Closely related genes such as *PIAL1* and *PIAL2*, *DeSI3a* and *DeSI3b* show strong clustering, thus suggesting a highly conserved gene expression response to the three stresses (fig. S15A). However, these same genes are not clustered tightly at the protein abundance level, indicating that the control of gene expression has diverged from the regulation of protein abundance during stress (fig. S15C). We next compared expression levels of all SUMO components both at the gene and protein expression levels against each other to identify clusters of genes that may be either positively or negatively correlated. One of the strongest clusters to come out of this analysis is one including *PIAL1*, *PIAL2*, *OTS2*, *DeSI4b*, *SPF2*, and *SUMO2*, which are all positively correlated on the basis of gene expression (fig. S15B). This might underlie a conserved functional link for these genes during a stress response. Protein cluster analysis demonstrated that SCE1, whose protein levels increased in all three stresses, negatively correlates with SAE1a, PIAL1, HPY2, and PIAL2 (fig. S15D). This suggests that SCE1-dependent SUMO conjugation to targets might be favored over polySUMOylation and HPY2-specific targets during stress responses.

### Different stresses trigger unique changes in the SCE1 interactome

The *Arabidopsis* genome contains a single SUMO E2 conjugation enzyme termed SCE1 (fig. S1). Our Cell Atlas data revealed SCE1 to be the only component of the SUMO system that is significantly increased under all three stresses (Figs. 3 to 5). Hence, we hypothesize that this E2 is the main driver modulating stress-dependent increases in SUMO conjugation (fig. S13).

**Fig. 6.**
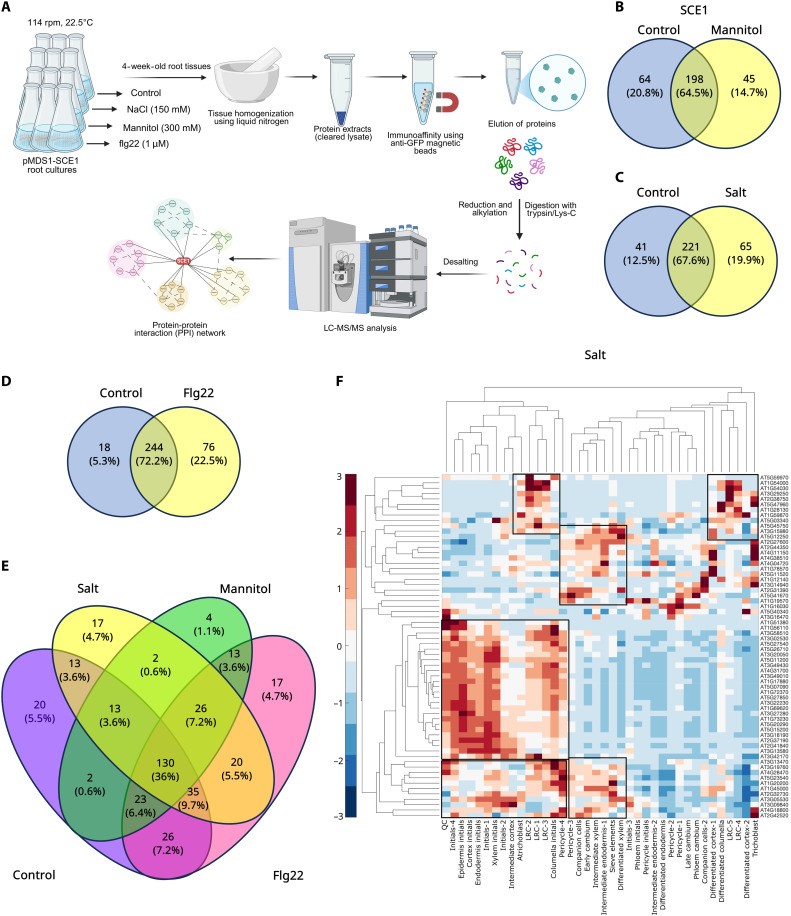
Stress-specific SCE1 interactome characterized by IP-MS analysis. (**A**) Schematic representation of the experimental design and proteomics workflow used for SCE1–IP-MS analysis under distinct stress conditions. (**B** to **D**) Venn diagrams illustrating overlapping and stress-specific SCE1 interactors identified under mannitol, salt, and flg22 treatments, respectively. (**E**) Venn diagram summarizing the overlap of SCE1 interactors across all tested stress conditions relative to the control. (**F**) Expression analysis of SCE1 interactors identified from the salt stress experiment across root tissues and their pseudotime trajectories. The heatmap displays the proportion *z*-score (−3 to 3) of standardized gene expression profiles across tissue types and zones. The red-blue colormap represents relative expression levels, with red indicating up-regulation (+1 to +3 SDs above the mean) and blue indicating down-regulation (−1 to −3 SDs below the mean), with the mean represented as 0. Black boxes highlight qualitative cluster groups, and dendrograms depict hierarchical clustering of genes (rows) and zones (columns) based on expression similarity.

We designed immunoprecipitation–mass spectrometry (IP-MS) experiments to identify SCE1 interacting partners under the three stresses to determine whether each treatment resulted in a distinct SUMOylated proteome. We performed these assays using root cultures expressing genomic SCE1 using the pMDS1 vector system (Fig. 1). The interactome pull-down assays were facilitated by the SCE1 protein being tagged at its C terminus with both 3xHA and mVenus tags. After treatment of the roots with NaCl (150 mM), mannitol (300 mM), or flg22 (1 μM) for 3 hours, IP was performed on extracted proteins using magnetic anti–green fluorescent protein (GFP) beads, and then the interactomes were analyzed using liquid chromatography–tandem MS (LC-MS/MS) analysis (Fig. 6A and fig. S16).

LC-MS analysis identified 262, 286, 243, and 320 putative interactors of SCE1-GFP-HA under control, salt, mannitol, and flg22 treatments, respectively (Fig. 6, B to D). Deeper analysis of the stress-specific interactors indicated that 65 were unique to salt, 45 to mannitol, and 76 to flg22 treatments compared to unstressed controls (Fig. 6, B to D, and data S1). Protein-protein interaction network analysis revealed that these stress-induced interacting targets are involved in various biological processes, highlighting their potential roles in response to distinct stress conditions (fig. S17). Of the stress-induced targets identified, 26 targets (26.3%) were common across different stress conditions, indicating that SCE1 interacts with these proteins regardless of the type of stress. In contrast, 17, 4, and 17 interactors were unique to salt, mannitol, and flg22 treatments, respectively (Fig. 6E), suggesting these proteins might shape stress-specific SUMOylated proteomes. A total of 70 of 361 interacting partners were identified as known targets of SUMO1 based on previous SUMOylome datasets (*7*, *31*, *32*). The remaining interactors identified under these conditions are less likely to be the SUMOylated targets themselves, as the pulldowns were performed under nondenaturing conditions, and hence, they may represent interacting partners of the SUMOylated targets. However, the interactors of SCE1 may shape how the SUMO E2 is regulated to drive the formation of SUMOylated protein. Given that SCE1 protein abundance changes in different cell types during these stresses, we ascertained where the interactors of SCE1 are predominantly expressed within the root cell types by cross-analysis of SCE1 imaging data against publicly available scRNA-seq data (*33*). This analysis reveals that under salt and mannitol stress, SCE1 interacts more with proteins expressed in epidermis and atrichoblast cells, while under flg22 treatment, it interacts predominantly with proteins expressed in the endodermis and xylem tissue (Fig. 6F and fig. S18). These data suggest that during abiotic stresses, the plant root SUMOylome is mainly derived from epidermis, while during flg22 treatment, the SUMOylome is generated within endodermis and xylem cells, suggesting that these inner tissues are the primary targets of the SUMO system for generating adaptive responses during biotic stress.

## DISCUSSION

### Cell Atlas reveals wide spatial variation in SUMO components

PTMs act at the core of biological processes by creating new proteoforms to transduce environmental cues into molecular responses. In this regard, they amplify the genomic potential of organisms by creating multiple functionally different variants from a single gene product. SUMO is an essential PTM in eukaryotes (*9*). Despite its importance in cell function, it has not been possible to visualize the activity of an entire SUMO system in multicellular organisms in response to external cues until now. Here, we describe the construction of a Cell Atlas for the entire *Arabidopsis* SUMO machinery, to our knowledge, the first of its kind in any eukaryote. To achieve this, we tagged all 32 components of the SUMO cycle involved in activating, conjugating, and removing the SUMO peptide. In doing so, we provide a resource for all researchers working on plant SUMOylation, which is located on the ePlant server (see Data and materials availability statement). This web application visualizes the expression and localization of all SUMO components in unstressed and stressed plants demonstrated as a heatmap. Seeds of all transgenic lines have been deposited in the Nottingham Arabidopsis Stock Centre. In addition, through the pMDS1 and pMDS2 vectors, we provide tools where others can simultaneously visualize gene expression and protein localization of their gene of interest. These resources will provide molecular, spatiotemporal, and regulatory information about the plant SUMO machinery that will allow researchers across different fields to explore the role of SUMOylation in a broad array of developmental programs and stresses and will be beneficial for a variety of abiotic and biotic stresses and those working in different tissues and different developmental stages.

Understanding how a PTM system is spatially resolved will provide fundamental information about how multicellular eukaryotes utilize proteoforms for cell signaling to produce an integrated response to changing environments. To our knowledge, this study represents the first time a whole PTM has been spatially resolved in any eukaryote, and this has provided unique insights into how the SUMO system is organized and what drives its specificity. In *Arabidopsis*, the E1 to E4 SUMO enzymes show broad expression across multiple cell types in the root tip. However, the E3 HPY2 and E4 enzymes PIAL1 and PIAL2 are enriched in the meristematic zone. On the basis of these proteins’ spatial specificity, a model is likely where SUMO is broadly conjugated to its targets across multiple cell types. Although most E1 to E4 enzymes of the conjugation system are predominantly nuclear localized, there is clear evidence that the E1 (SAE complex) and E2 (SCE1) are present in significant levels in the cytoplasm, suggesting that protein SUMOylation outside the nuclear compartment is mainly driven by SCE1.

The SUMO peptides show considerable variation in their cell-type–specific patterns. The *Arabidopsis* genome contains eight SUMO paralogs (*34*, *35*). Five of these showed expression in the root tip, specifically *SUMO1*, *SUMO2*, *SUMO3*, *SUMO4*, and *SUMO5*, while no expression was found in *SUMO6*, *SUMO7*, and *SUMO8*. SUMO1 and SUMO2 are thought to be the main drivers of SUMOylation in plants, as they demonstrate the highest levels of expression (*36*), and the *sumo1-1 sumo2-1* double knockout is embryo lethal (*18*). SUMO1 and SUMO2 share an 83% amino acid sequence similarity, whereas SUMO1 and SUMO3, SUMO4, and SUMO5 share considerably less (46, 36, and 37%, respectively). In the processed mature forms, SUMO1 and SUMO2 are so similar that MS-based analysis for SUMO-conjugated targets may not be able to distinguish between these isoforms, causing researchers to assume that both genes were broadly expressed and function in a similar manner. Here, we demonstrate a clear distinction in spatial expression domains. SUMO2 displays a broad zone of expression and protein abundance within root tip tissues, while SUMO1 is expressed in phloem pole companion cells starting from the root EZ. The contrasting expression pattern of *SUMO1* and *SUMO2* would enable conjugation to be controlled in a cell-type–specific manner under inductive conditions. However, *sumo1-1* and *sumo2-1* single mutants display no obvious phenotypic defects in unstressed conditions (*18*, *37*), suggesting that they normally act redundantly. Hence, the phenotypes of these single-mutant lines await characterization under a range of stress conditions to reveal SUMO1- and SUMO2-specific roles.

Other SUMOs expressed in roots (3, 4, and 5) likely play more specialized roles in specific tissues. Of these three, only *sumo3-1* mutants have been studied to date, revealing a small delay in flowering time and a role in plant defense response downstream of salicylic acid (*37*). Unlike SUMO1/2, SUMO3 cannot form poly-SUMO chains and likely has unique target substrates (*38*). Expression of SUMO3 primarily in the epidermis is in accordance with its role in plant defense during root growth in soil. SUMO4 was previously thought not to be expressed in root tissue (*34*). Still, our study reveals weak expression in the late elongation and maturation zone tissues, while SUMO5 localizes strongly to the stele and meristematic epidermal cells. To our knowledge, this is the first observation of SUMO5 tissue and cell-specific expression in plants. The high level of spatial variation in expression between the SUMOs suggests that SUMO2 drives the main developmental SUMO system, as it is widely expressed. In contrast, cell-type–specific expression of *SUMO1*, *SUMO3*, *SUMO4*, and *SUMO5* allows for fine-tuning of SUMO-mediated responses in a tissue or cell-type–specific manner.

Our SUMO Cell Atlas also revealed that DeSI proteases exhibit variable subcellular localization and cell-type specificity. This class of SUMO protease was first identified in mammalian cells as an evolutionarily distinct group from ULP proteases (*39*, *40*). DeSIs have isopeptidase activity, deconjugating SUMO from substrates, but cannot process SUMO, whereas the ULP class of proteases catalyzes both processes. ULP proteases are widely expressed in different cell types and localize largely to the nucleus, potentially explained by their critical role in SUMO maturation. In plants, the only non–nuclear-localized SUMO protease to be described to date was DeSI3a, which localizes to the plasma membrane (*21*). This localization is key to its role in deSUMOylation of the membrane-bound FLS receptor. Our current study reveals that the DeSI family exhibits great variability in subcellular localization, ranging across the cytoplasm, plasma membrane, and vacuolar membranes. DeSI3a, DeSI3b, and DeSI3c are all membrane localized; however, the mVenus signal in DeSI3a displays speckles on the membrane, suggesting highly specific binding to a select group of membrane proteins. This indicates the DeSIs control deSUMOylation of a small subset of substrates locally involved in fine-tuning stress responses. In contrast, ULP proteases appear to play a more general role in SUMO maturation and nuclear processes across multiple root tissues. Collectively, these data show subfunctionalization of the SUMO signaling machinery often at a subcellular scale, providing a basis for individual stress responses to modulate bespoke downstream processes.

### Stress-specific modulation of the SUMO system

SUMOylation represents a vital PTM to modify cellular targets in response to changing environmental signals and stresses. In this study, we tested how the SUMO system in *Arabidopsis* root tissues reacted to three model environmental stresses: salt, osmotic, and biotic signals. Each model stress triggers a distinct response in terms of the SUMO components being regulated and which root tissues respond. For example, each of the stresses regulates SUMO modifiers. Osmotic stress causes levels of SUMO2 and SUMO3 to increase, and under salt stress, SUMO2 levels go down, while after flagellin treatment, only SUMO3 levels increase in epidermal cells. Our results also reveal that biotic stress targets much fewer components of the SUMO system compared to salt and osmotic stresses, which induce marked changes in the transcriptional and translational status of most of the SUMO system components. Nevertheless, our observations reveal that the SUMOylated proteome in response to different stresses is shaped mostly by SUMO2 and SUMO3. The SUMO system distinguishes between different abiotic stresses at the tissue-specific level and cell-specific level to generate spatially resolved SUMOylated proteomes. This evidence hints at the possibility of these root tissues being key for enacting adaptive responses mediated by SUMO. Isolating tissue-specific SUMOylomes promises to unlock the molecular pathways that integrate root growth and development with its changing environment.

It had previously been documented that SUMO conjugates accumulate upon different stresses in eukaryotes. Our Cell Atlas has allowed us to demonstrate that, in plant roots, this increase in SUMO conjugation is primarily driven by an increase in protein abundance of SCE1, the E2-conjugating enzyme, rather than E3s. The SCE1 interactome supports this observation, as distinct interacting partners were observed for each stress applied. We postulate that specific tissues respond by increasing SCE1 levels and its interactome to shape the SUMOylated proteome upon stress perception. A tissue-specific change in the SUMOylated proteome landscape enacts the adaptive responses we observe, such as hydropatterning in roots (*30*). The Cell Atlas provides researchers with resources to extend this observation beyond the root to ascertain whether SCE1 is also the principal driver of increased SUMO conjugation in other plant organs, developmental processes, and stress responses.

SUMO modification status reflects the balance between the activity of the E1 to E4 conjugation system and deconjugation controlled by ULP and DeSI classes of SUMO proteases. Gene families of these SUMO proteases have expanded, as plants evolved to adapt to diverse environmental conditions on land, while the numbers of genes encoding the E1 to E4 conjugation system remained unexpanded (*9*). Our Cell Atlas revealed unique combinations of SUMO protease expression changes in response to each of the three stresses. For example, following flg22 elicitor treatment, OTS1 and DeSI3a were specifically turned over, while ESD4, DeSI2b, and DeSI3C increased in abundance. This observation suggests that the flg22-dependent SUMOylome is shaped by the increase in SCE1-dependent conjugation and ESD4, DeSI2b, and DeSI3c deconjugation, followed by a decrease in OTS1 and DeSI3a deconjugation. In contrast, salt stress reduced OTS1 and OTS2, DeSI1, and DeSI4a abundance, while SPF1 and ELS1 increased in a tissue-specific manner. This insight into the tissue/cell-specific SUMO “coding” system opens up avenues for identifying stress-specific SUMOylated targets. For example, osmotic stress–dependent SUMOylation of the transcription factor AUXIN RESPONSE FACTOR7 (ARF7) negatively regulates its DNA binding activity through recruitment of the Aux/IAA (indole-3-acetic acid) repressor protein IAA3 (*34*), and this, in turn, represses lateral root development during osmotic stress. The atlas revealed that the main driver of ARF7 SUMOylation is likely to be a combinational outcome of enhanced SCE1 expression and repression of the SUMO protease OTS1 that has previously been reported to target ARF7 for deSUMOylation. However, the expression of SUMO proteases FUG1, SPF1, and DeSI3a is also repressed during osmotic stress, but their role in lateral root development has not been established, and opens up avenues for further research.

There is correlative evidence for the neofunctionalization and gene expansion of SUMO proteases, through fusion of conserved catalytic domains with divergent sequences with the emergence of adaptive traits in the plant lineage (*9*). Our data indicate that a major driver of specificity within the SUMO system in plants emanates from the spatial regulation of the SUMO proteases.

## MATERIALS AND METHODS

### Generating transgenic material

The generation of polycistronic vectors (pMDS1 and pMDS2) that enables simultaneous monitoring of both the transcription and translation of a gene via confocal imaging and Western blot involved several cloning steps. For the pMDS1 (C-terminal fusion), first, the Venus fluorescence coding sequence was PCR amplified (primers 863/864 and template DR5:Venus:N7) and inserted into a pGWB13 vector containing a 3xHA tag and Xba/PacI sites using Hot Fusion cloning (table S1). From this vector, the VENUS:3xHA sequence was amplified by PCR with the addition of a long glycine-serine linker sequence on the 5′ end. This PCR product was cloned into the HindIII site of pMCY2, a vector containing mCherry for seed selection and a cell imaging marker. Next, the 2A:mTurquoise (primers 868/79) and N7 (primers 869/870 and template DR5:Venus:N7) coding sequences were separately amplified by PCR and simultaneously cloned into the pMCY2 vector containing the GSlinker:Venus:3xHA sequence using Hot Fusion into the Bsu36I site. The mTurquoise was reeducated to remove a SapI restriction site of its sequence (primers 874, 875, 876, and 877) and reinserted into the vector using HindIII digestion and Hot Fusion reaction. The final plasmid containing GSlinker:Venus:3xHA:2A:mTurquoise:N7 was named pMDS1.

For generation of the pMDS2 (N-terminal fusion), we amplified 3xHA from pMDS1 using primers 893 and 867, followed by insertion via Hot Fusion of the gel-purified fragment into a HindIII-digested pMCY2 (table S1). The resulting vector was then linearized at a BSU36I site (introduced by primer 867) to enable insertion of the GSG-Linker:mVenus fragment, which was amplified from pMDS1 using primers 880 and 881. Subsequently, this construct was linearized at the BSU36I site (introduced by 881) to allow insertion of the 2A peptide–mTurquoise fragment, which was amplified from pMDS1 using primers 886, 888, and 870. The final pMDS2 plasmid contains a single BSU36I site that can be used to generate an N-terminal 3xHA:GSG:Venus fusion protein.

### Making of the pMDS1and pMDS2 DNA constructs

Gene-specific primers were designed to clone the promoter (~2 kb or up to the previous open reading frame in cis) and genomic region of the 32 SUMO components into the pMDS1 or pMDS2 vector using the Hot Fusion approach (table S2) (*41*), at SapI sites for pMDS1 and SapI and Bsu36I sites for pMDS2. For the ELS1 construct, a shorter promoter was used (500 base pairs), as the 2-kb promoter did not express. The final plasmid was transformed into *Agrobacterium tumefaciens GV3101* strain containing the pSOUP helper plasmid by electroporation and then transformed into *A. thaliana* ecotype Col-0 by the floral dip method for generating Cell Atlas transgenics. For complementation lines, the following mutant backgrounds were used: *sum3-1* (SALK_123673C) and *siz1-3* (SALK_034008). The seedlings were grown at 21°C. The growth conditions for individual experiments are listed in the “Plant materials and growth conditions” section below.

### Plant materials and growth conditions

Seeds were surface sterilized for 4 min in 30% (v/v) bleach containing 0.005% Triton X-100, followed by five washes with sterile water and then stratified at 4°C for 48 hours in the dark. Seeds were germinated on a medium containing half-strength (^1^/_2_) Murashige and Skoog medium (Sigma-Aldrich), 0.4% sucrose, and 1% Bacto-agar at pH 5.7. Seedlings were grown vertically for 5 to 6 days under a day temperature of 23°C and a night temperature of 18°C with a 16-hour photoperiod (150 μmol m^−2^ s^−1^). All transgenic material and constructs are listed in table S3.

### Confocal settings for SUMO Cell Atlas

T3 plants of the different SUMO components were imaged using a Leica SP8 confocal microscope (Leica Microsystems) with a 20× objective and sequential scanning using Tile Scan with automated merging (Fig. 2). The mTurquoise signal was excited at 422 nm with a 422-nm diode and light captured between 452 and 505 nm using a hybrid GaAsp/APD (HyD). For the mVenus signal, we used an Argon laser at 516 nm and light captured between 520 and 570 nm using a HyD. The mCherry signal was excited with a 561-nm diode-pumped solid-state (DPSS) laser, and light between 590 and 731 nm was collected using two photomultiplier tube detectors. To understand the relative expression of these variably expressed components, three laser powers for both the 422-nm diode and the argon laser were used: 2, 14, and 26%, while the gain stayed the same (150 V).

To create the split images, Tile Scan images were used, and roots were straightened with Fiji’s straighten function. This allowed us to make clear splits through the longitudinal middle of each root. One-half of the root was then chosen, in which the nuclei line up in the different cell types in the mTurquoise channel. This half was then split into three channels, and afterward, mTurquoise and mCherry were fused back together. One-half would then be flipped horizontally to mirror the other half.

For the subcellular SUMO Cell Atlas, we used a ×63 water immersion objective and, otherwise, the same laser settings. The laser powers were adjusted on the basis of the fluorescence level of the reporter line.

### SUMO Cell Atlas analysis

Fiji software was used for image analysis. As three different settings were used to capture the extent of the variety of fluorescence signals, we divided the relative level of fluorescence into six different bins (table S4).

### Confocal settings for stress atlas

For imaging the SUMO stress atlas, the same confocal settings as for Fig. 2 were used, but instead of imaging in three different laser powers, only the optimum was chosen per gene, and this was not changed during the experiment and repeats (Figs. 3 to 5). Experiments were repeated twice with eight roots per treatment. The median experiment of three and the four median roots within that experiment were chosen for detailed tissue-specific analysis. This was based on whole-image fluorescence of both the mTurquoise and mVenus channels.

### Mannitol stress

Six-day-old *Arabidopsis* seedlings were transferred from ^1^/_2_ MS plates to plates either containing 300 mM mannitol or mock plates. After 3 hours, seedlings were mounted on a slide and scanned on the confocal microscope.

### Salt stress

Five-day-old *Arabidopsis* seedlings grown on ^1^/_2_ MS medium were transferred to a setup containing liquid ^1^/_2_ MS supplemented with 150 mM NaCl, ensuring that only the roots were exposed to the salt treatment while the aerial parts remained untreated. After 3 hours of salt exposure, the seedlings were mounted on slides and imaged using a confocal microscope. A parallel experimental setup containing only liquid ^1^/_2_ MS was used as a reference control.

### Flagellin stress

Five-day-old *Arabidopsis* seedlings grown on ^1^/_2_ MS medium were transferred to a setup containing liquid ^1^/_2_ MS supplemented with 1 μM fgl22, ensuring that only the roots were exposed to the salt treatment while the aerial parts remained untreated. After 3 hours of salt exposure, the seedlings were mounted on slides and imaged using a confocal microscope. A parallel experimental setup containing only liquid ^1^/_2_ MS was used as a reference control.

### Stress atlas and tissue-specific analysis

Fiji software was used for the tissue-specific data analysis. A box was drawn around the four tissue types measured: epidermis, cortex, endodermis, and stele. Raw fluorescence was measured and divided by the area of measurement to account for differences in area size. Fluorescence in two zones was measured—meristem and EZ. The meristem zone was defined by the point where the cortex cell becomes twice the width in length. The EZ is measured from the end of the meristem to the start of root hair bulging. The measured fluorescence under stress versus control conditions was compared for significance using a paired *t* test with the Bonferroni method to adjust the *P* values. For each tissue type, the number of sumo system genes that show significantly different levels in their transcript or protein abundance was counted on the basis of their *P* value and the log_2_ fold change under stress versus control, i.e., up-regulated when log_2_ fold change > 0 and *P* < 0.05, down-regulated when log_2_ fold change < 0 and *P* < 0.05, and unchanged for *P* ≥ 0.05.

### RNA isolation and qRT-PCR

For the quantification of the expression level of endogenous and exogenous genes in the reporter lines, roots of 6-day-old seedlings (~100 roots per sample) were harvested. Total RNA was extracted using the RNeasy Plant Mini Kit (QIAGEN). Total level of RNA was then measured using the NanoDrop 1000 Spectrophotometer (Thermo Fisher Scientific) and normalized before cDNA synthesis. cDNA was synthesized using the Invitrogen SuperScript II Reverse Transcriptase. Forward primers for qRT-PCR of endogenous gene expression were designed, when possible, to span an exon junction so as to avoid amplification of contaminated genomic DNA. The reverse primer was designed using the 3′ untranslated region, as this was not included in the construct design. Exogenous gene expression primers used the Venus-N7 region as an indication of transgene expression level. A full list of primers is shown in table S5. qRT-PCR was performed using the Analytik Jena qTower^3^ 84 G Real-Time PCR System (Nottingham) and the QIAGEN Rotor-Gene Q system (Durham). Actin2 (AT3G18780) was used as a housekeeping gene in case of high expression. Low gene expression used protein phosphatase 2A as a housekeeping gene. Three biological replicates and three technical replicates were used in this experiment. The difference (ΔCt) in the expression of the transgene or the endogenous gene was calculated with reference to the housekeeping gene and is plotted as 2^–(ΔCt)^. The significance between the expression of trans- and endogenous genes was calculated using a Student’s *t* test; *P* < 0.05.

### RNA isolation (for bulk RNA-seq)

For the RNA-seq stress dataset, roots of 6-day-old seedlings (~120 roots per sample and three samples per treatment) were harvested 3 hours after stress treatment. Total RNA was extracted using the RNeasy Plant Mini Kit (QIAGEN). Extracted RNA was sent to Novogene Europe where a polyadenylated nonstranded library prep was done, followed by Illumina PE150 sequencing to a 3-gigabase depth conducted on the NovaSeq 6000 S4 platform.

### Western blotting

*A. thaliana* seedlings of the appropriate reporter line were treated with salt, mannitol, or flagellin for 3 hours, after which 50 roots were harvested and flash frozen in liquid nitrogen. Total protein was extracted using Laemmli buffer containing 50 mM tris-HCl (pH 6.8), 2% SDS, 10% glycerol, 147 mM B-mercaptoethanol, 12.5 mM EDTA, and 2 mg bromophenol blue made up in deionized water. After protein extraction, the samples were separated by SDS–polyacrylamide gel electrophoresis (SDS-PAGE) and blotted onto a polyvinylidene difluoride (PVDF) membrane. The PVDF membrane was then blocked with 5% (w/v) skimmed milk in 1× tris-buffered saline with 0.1% Tween 20. The membrane was then either probed with an anti-HA antibody (3F10, Roche) or anti-UGPase (Agrisera) antibody, followed by anti-Rat horseradish peroxidase (HRP; Abcam) or anti-Rabbit HRP (Abcam). The membranes were treated with SuperSignal West Pico PLUS Chemiluminescent Substrate (Thermo Fisher Scientific), and the luminescence was detected using an x-ray cassette and a fixer and developer. UGPase levels in untreated and treated samples were used as loading reference controls.

### IP assay for assessing effective 2A peptide skipping

To assess the level of efficiency in mediating ribosomal skipping by the 2A peptide in the pMDS1/pMDS2 vector system, we performed IP assays using anti-GFP magnetic beads (Miltenyi Biotech). *A. thaliana* seedlings were grown in Gamborg B5 medium with added B5 vitamin and sucrose. Roots were harvested, and total protein was extracted using the following extraction buffer (EB): 50 mM tris-HCl (pH 8.5), 150 mM NaCl, 1 mM EDTA, 0.1% (v/v) SDS, 0.5% (w/v) sodium deoxycholate, 1.0% (v/v) IGEPAL CA-630 (Nonidet P-40), 50 mM KCl, 50 mM *N*-ethylmaleimide, and a protease inhibitor cocktail (one tablet per 10 ml of buffer). Total protein extract was incubated with anti-GFP beads (Miltenyi Biotech) on a rotator at 4°C at a speed of 20 to 25 rpm for 30 min. Afterward, the extract was loaded into a micro column (Miltenyi Biotech) in a magnetic stand. Beads were then washed four times with ice-cold IP EB. Eight microliters of preheated (98°C) Laemmli buffer was used to elute the protein. The eluted immunoprecipitated fraction was lastly probed with anti-GFP antibody (Abcam, ab6556) to detect the various forms of the target fusion protein and the transcriptional reporter mTurquoise. Efficient ribosomal skipping induced by the 2A peptide will result in two polypeptides that include POI-GFP-3XHA and N7-mTurquoise, where POI stands for protein of interest.

### IP for SCE1 interactors

Proteins were extracted from the root tissues of pMDS1-SCE1 plants using three biological replicates for IP experiments. As a negative control, root tissues from pMDS1-VAM3 and pMDS1-SHR were used to account for nonspecific binding to GFP, as well as to exclude interactions with SUMO pathway–unrelated proteins, such as VAM3 and SHR. The protein EB contained 50 mM tris-Cl (pH 7.5), 125 mM NaCl, 1.5 mM MgCl2, 1 mM EDTA, 5% (v/v) glycerol, 0.1% (v/v) Tween 20, 0.2% (v/v) IGEPAL CA-630, and 0.5% (w/v) digitonin, supplemented with freshly added protease inhibitors (cOmplete Mini EDTA-free, Roche; 1 tablet per 10 ml of solution). Root tissues (3 g) were ground to a fine powder under liquid nitrogen using a mortar and pestle. The powdered tissue was mixed with 6 ml of EB, and the lysate was clarified by centrifugation at 14,000*g* for 10 min.

The supernatants were incubated with 50 μl of anti-GFP magnetic beads (Miltenyi Biotec, catalog no. 130-091-125) on an end-to-end rotator at 4°C for 2 hours. Bead-bound lysates were passed through microcolumns (Miltenyi Biotec, catalog no. 130-042-701) mounted on the μMACS Separator. The beads were washed three times with chilled IP wash buffer (EB without digitonin), and the immunocomplexes were eluted using 50 μl of preheated (95°C) 1× SDS-loading buffer. Eluted proteins were resolved on 4 to 15% gradient SDS-PAGE gels and analyzed by immunoblotting with an anti-GFP antibody (Abcam, ab6556) to confirm SCE1 enrichment.

The immunoprecipitated eluates were further resolved on a 12% SDS-PAGE gel, electrophoresed for 20 min to allow protein entry into the resolving gel, and stained with colloidal Coomassie brilliant blue. Destaining was performed with a solution containing 50% water, 45% methanol, and 5% glacial acetic acid for 30 min. Protein bands were excised into ~1-mm^3^ pieces using a sterile blade, transferred to 1.5-ml tubes, and washed three times with a solution of 50% acetonitrile (ACN) and 50 mM ammonium bicarbonate (ABC) to remove the stain. The gel pieces were dehydrated in 100% ACN for 10 min. Disulfide bonds were reduced with 10 mM dithiothreitol in 50 mM ABC at 37°C for 1 hour, followed by alkylation with 55 mM iodoacetamide in 50 mM ABC at room temperature in the dark for 45 min. The gel pieces were again dehydrated with 100% ACN for 10 min.

After removing residual ACN, the gel pieces underwent in-gel digestion with trypsin/Lys-C mix (Promega, V5071) at a protease-to-protein ratio of 1:25 (w/w) at 37°C for 16 hours. Peptides were extracted in 0.1% formic acid (FA), dried in SpeedVac, and desalted using Pierce Peptide Desalting Spin Columns (Thermo Fisher Scientific, catalog no. 89851) following the manufacturer’s protocol. Desalted peptides were dissolved in 0.1% FA for analysis using a Q Exactive Orbitrap Mass Spectrometer.

### LC-MS/MS analysis

All LC-MS/MS experiments were performed using a Dionex UltiMate 3000 RSLCnano UPLC (Thermo Fisher Scientific, Waltham, MA, USA) system and a Q Exactive Orbitrap Mass Spectrometer (Thermo Fisher Scientific, Waltham, MA, USA). Separation of peptides was performed by reverse-phase chromatography at a flow rate of 300 nl/min and a Thermo Fisher Scientific reverse-phase nano EASY-Spray column (PepMap C18, Thermo Fisher Scientific; 2-μm particle size, 100-Å pore size, 75-μm inner diameter × 50-cm length). Five hundred nanograms of peptides was loaded onto a precolumn (PepMap 100 C18, Thermo Fisher Scientific; 5-μm particle size, 100-Å pore size, 300-μm inner diameter × 5-mm length) from the UltiMate 3000 autosampler with 0.1% FA for 3 min at a flow rate of 15 μl/min. After this period, the column valve was switched to allow elution of peptides from the precolumn onto the analytical column. Solvent A was water + 0.1% FA, and solvent B was 80% ACN and 20% water + 0.1% FA. The linear gradient used was 2 to 40% B in 90 min. Further wash and equilibration steps gave a total run time of 120 min.

The LC eluant was sprayed into the mass spectrometer by means of an EASY-Spray source (Thermo Fisher Scientific). All mass/charge ratio (*m/z*) values of eluting ions were measured in an Orbitrap mass analyzer, set at a resolution of 35,000, and were scanned between *m/z* of 380 and 1500. Data-dependent scans (top 20) were used to automatically isolate and generate fragment ions by higher-energy collisional dissociation (HCD; normalized collision energy (NCE): 26%) in the HCD collision cell, and measurement of the resulting fragment ions was performed in the Orbitrap analyzer, set at a resolution of 17,500. Singly charged ions and ions with unassigned charge states were excluded from being selected for MS/MS, and a dynamic exclusion window of 20 s was used.

The acquired MS/MS spectra were analyzed using Mascot against the *Arabidopsis* protein database (uniprotkb_proteome_UP000006548_2024_10_18.fasta) to identify potential SCE1 interactors. The Venn diagrams were generated using the Venny 2.1 online tool (https://bioinfogp.cnb.csic.es/tools/venny/). Protein-protein interaction networks were constructed using the STRING database (https://string-db.org), and GO analysis of stress-specific SCE1 interactors was performed using the ShinyGO 0.81 online tool (http://bioinformatics.sdstate.edu/go/). *A. thaliana* root scRNA-seq data (*42*) were used to extract the expression values of SCE interactors identified from mannitol, salt, and flagellin treatments across different cell clusters. The cell cluster (cell lineages or cell types) and their pseudotime trajectory nomenclature were followed as specified in Plant sc-Atlas (https://bioit3.irc.ugent.be/plant-sc-atlas/), with each expression value being the average expression value of that gene within the specified cell cluster. The data matrix for each treatment was organized, with rows representing individual genes and columns representing cell clusters (see cell cluster names in Fig. 6F). The dataset was standardized across rows to center the data and ensure uniform scaling, thereby enabling comparison between different cell clusters (see scale bar in Fig. 6F). MATLAB’s built-in clustergram function was used to perform hierarchical clustering on both rows and columns, using the default distance metric and linkage method. The resulting clustergram was visualized as a heatmap with dendrograms to depict the hierarchical relationships.

### Bulk RNA-seq analysis and statistics

For bulk RNA-seq analysis, the pipeline was adapted from Sriden and Charoensawan (*43*). The FASTQ files were quality control checked by FastQC (www.bioinformatics.babraham.ac.uk/projects/fastqc/), and the adaptors and low-quality reads were removed by Trimmomatic (*44*). High-quality reads were then mapped to Araport11 (*45*), the duplicated reads were discarded by Picard (https://broadinstitute.github.io/picard/), and the read abundances were quantified by HTSeq (*46*). DEGs were analyzed by DESeq2 (*47*). The transcripts that were expressed at *P* < 0.05 and fold change ≥ 1 and ≤ −1 were considered significantly up- and down-regulated DEGs.

### Single-cell sequencing analysis

scRNA-seq datasets of wild-type *Arabidopsis* root samples under control conditions were used from the following studies: (GSE123013) (*48*), (GSE123818) (*49*), and (GSE152766) (*50*). A total of 15 samples (except sc_9 and sc_10 from GSE152766) were chosen for further analysis. Individual biological samples from Denyer *et al.* (*49*) and Shahan *et al.* (*50*) were integrated into the respective Seurat objects (https://doi.org/10.1016/j.cell.2021.04.048) (*51*). Scaled values from the respective Seurat objects for each SUMO gene were used for comparisons.
