# Supplementary material for: Elucidating tissue and subcellular specificity of the entire SUMO network reveals how stress responses are fine-tuned in a eukaryote
Source: Sci Adv. 2025 Aug 27;11(35):eadw9153. doi: 10.1126/sciadv.adw9153 (PMC12383270; doi:10.1126/sciadv.adw9153)
Supplement: Supplementary file 1 — Figs. S1 to S18 Tables S1 to S5 Legend for data S1 [file sciadv.adw9153_sm.pdf]

Science Advances
AAAS

Supplementary Materials for

Elucidating tissue and subcellular specificity of the entire SUMO network reveals how stress responses are fine-tuned in a eukaryote

Jason Banda *et al.*

Corresponding author: Ari Sadanandom, ari.sadanandom@durham.ac.uk;
Anthony Bishopp, anthony.bishopp@nottingham.ac.uk; Miguel De Lucas, miguel.de-lucas@durham.ac.uk;
Shraboni Ghosh, shraboni.ghosh@durham.ac.uk; Jason Banda, jason.banda2@nottingham.ac.uk;
Dipan Roy, dipan.roy@durham.ac.uk

Sci. Adv. **11**, eadw9153 (2025)
DOI: 10.1126/sciadv.adw9153

The PDF file includes:

Figs. S1 to S18
Tables S1 to S5
Legend for data S1

Other Supplementary Material for this manuscript includes the following:

Data S1

A)

Type	Symbol	TAIR ID
SUMO modifier	SUMO 1	At4g26840
	SUMO 2	At5g55160
	SUMO 3	At5g55170
	SUMO 4	At5g48700
	SUMO 5	At2g32765
	SUMO 6	At5g48710
	SUMO 7	At5g55855
	SUMO 8	At5g55856
E1 activating	SAE1a	At4g24940
	SAE1b	At5g50580 /At5g50680
	SAE2	At2g21470
E2 ligase	SCE1	At3g57870
E3 ligase	SIZ1	At5g60410
	HPY2	At3g15150
E4 ligase	PIAL 1	At1g08910
	PIAL 2	At5g41580
SUMO protease	OTS1	At1g60220
	OTS2	At1g10570
	ESD4	At4g15880
	ELS1	At3g06910
	ELS2	At4g00690
	FUG1	At3g48480
	SPF1	At1g09730
	SPF2	At4g33620
	Desi1	At3g07090
	Desi2a	At4g25660
	Desi2b	At4g25680
	Desi3a	At1g47740
	Desi3b	At2g25190
	Desi3c	At5g25170
	Desi4a	At4g17486
	Desi4b	At5g47310

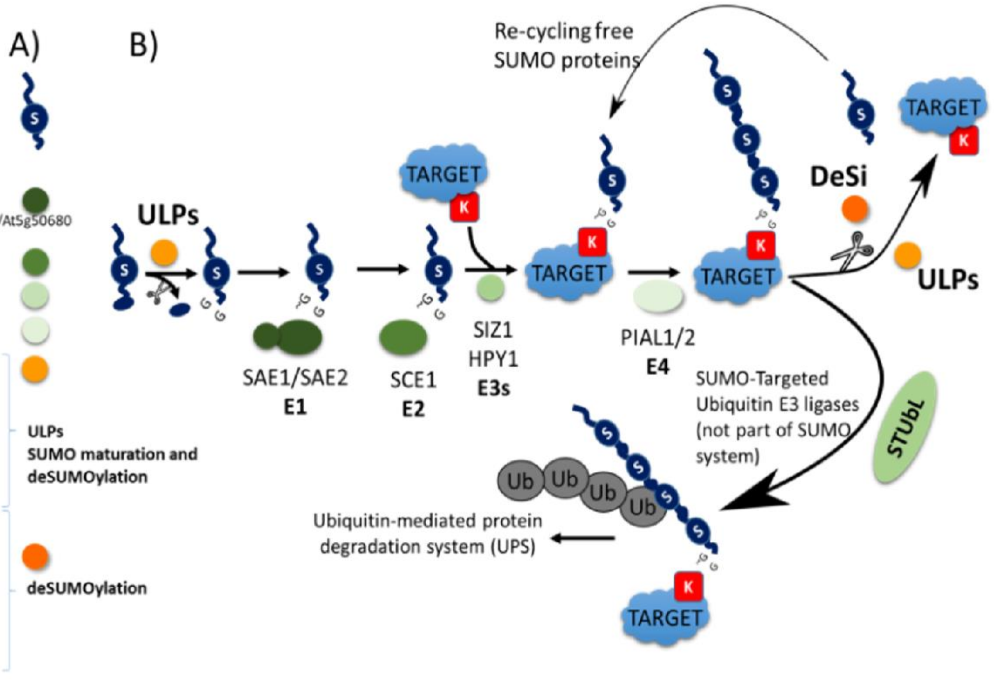

Fig. S1. The SUMOylation cycle and all known genes in Arabidopsis thaliana.

(A) Table of known SUMO machinery genes in *Arabidopsis thaliana*; (B) Schematic of SUMO System composition and functions in *Arabidopsis thaliana*. The ULP class of SUMO proteases process the precursor SUMO modifier at the C-terminus to expose the diglycine C-terminal end (-GG). The E1 (2 subunits; SAE1/SAE2) initiates the enzymatic conjugation cascade of the SUMO modifier, resulting in the conjugation of the SUMO modifier to the lysine (K) residues on substrates. ULP and DeSI class of SUMO proteases cleave SUMO off-targets. ULP= UBL-specific proteases, (UBL= Ubiquitin-like family protein modifiers); DeSI = DeSumoylating Isopeptidase; STUbl =SUMO-targeted ubiquitin ligases; Ub=Ubiquitin.

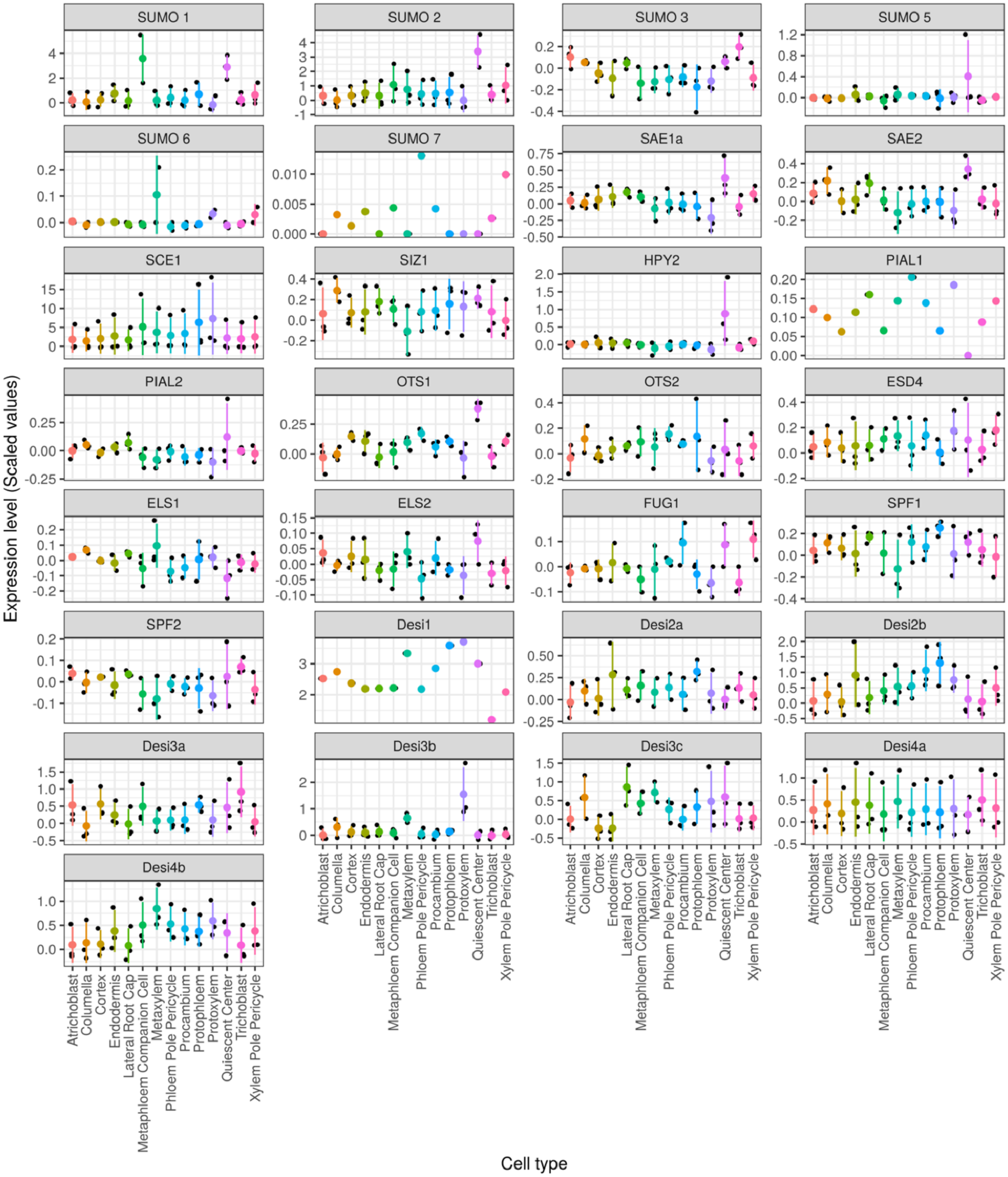

Fig. S2. **Expression levels of candidate genes representing the Arabidopsis SUMO machinery across various root cell types.**

The figure shows the expression levels of SUMO system genes from scRNAseq experiments across multiple cell types of the Arabidopsis roots. Each plot's coloured bold dot and line represent the mean and standard deviation. Black dots for each cell type are the scaled and transformed expression values from 3 scRNAseq datasets.

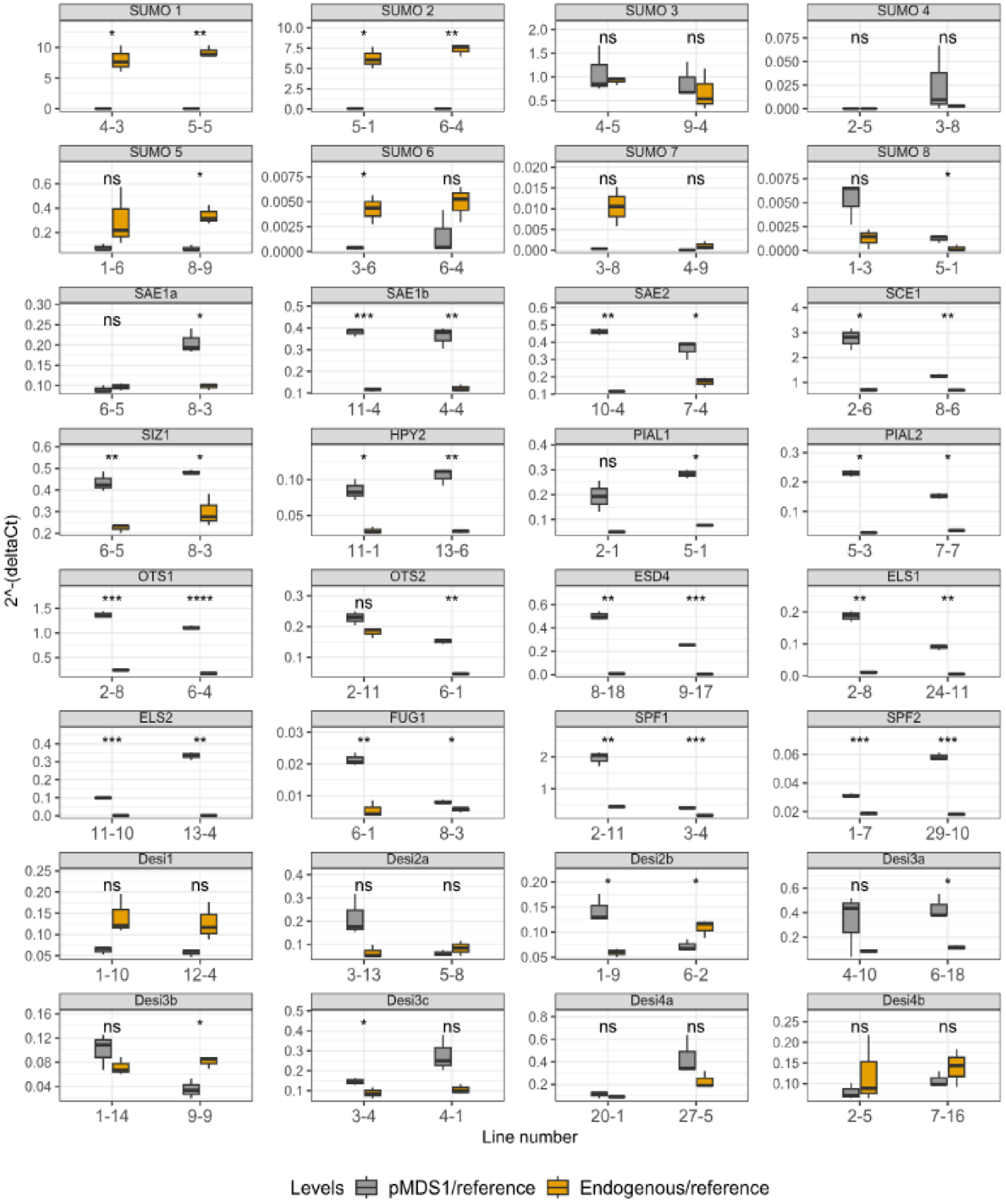

Fig. S3. **Expression levels of candidate genes representing the Arabidopsis SUMO machinery across independent transgenic lines developed for this study.**

Quantitative real-time PCR analysis was performed to assess the gene expression levels of the transgene (incorporated in the pMDS vector) in comparison to its endogenous counterpart in the transgenic lines of All 32 SUMO components. Three biological replicates and three technical replicates were used in this experiment. The plotted values show the $2^{-(\Delta Ct)}$ values for the endogenous gene vs pMDS1-fusion gene expression against the respective reference genes. The statistical significance of the difference between pMDS1 and endogenous gene as indicated by Student's t-test is shown by the asterisk (*). '****', '***', '**', '*', and 'ns' represent a p-value cut-off of value < 0.0001, < 0.001, < 0.01, < 0.05, > 0.05, respectively.

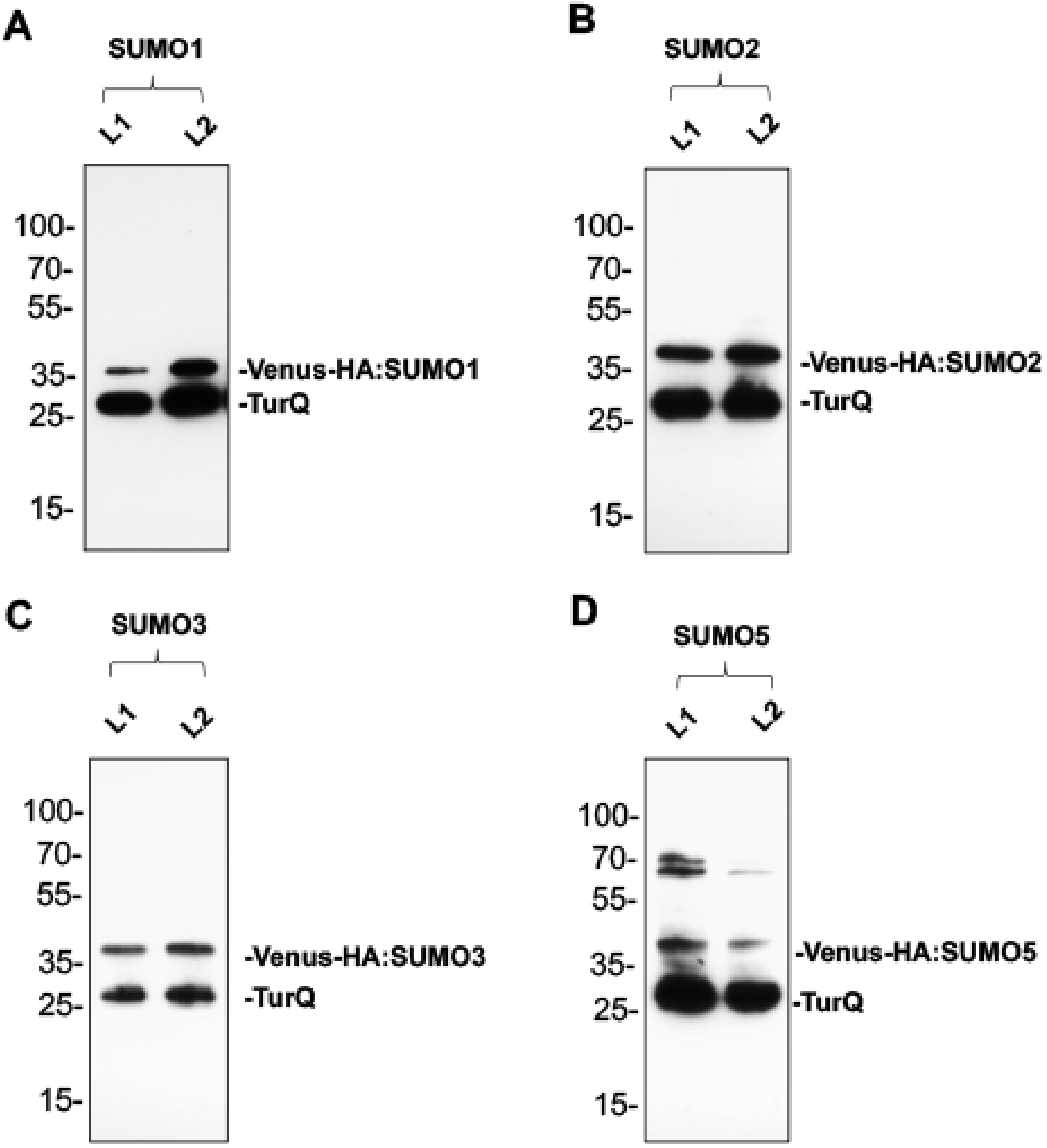

Fig. S4. Immunoblots of the SUMO modifier expressing pMDS2 lines indicating efficient ribosomal skipping induced by the 2A peptides.

(A) SUMO1, (B) SUMO2, (C) SUMO3 and (D) SUMO5. Bands corresponding to mTurquoise are labelled as mTurq.

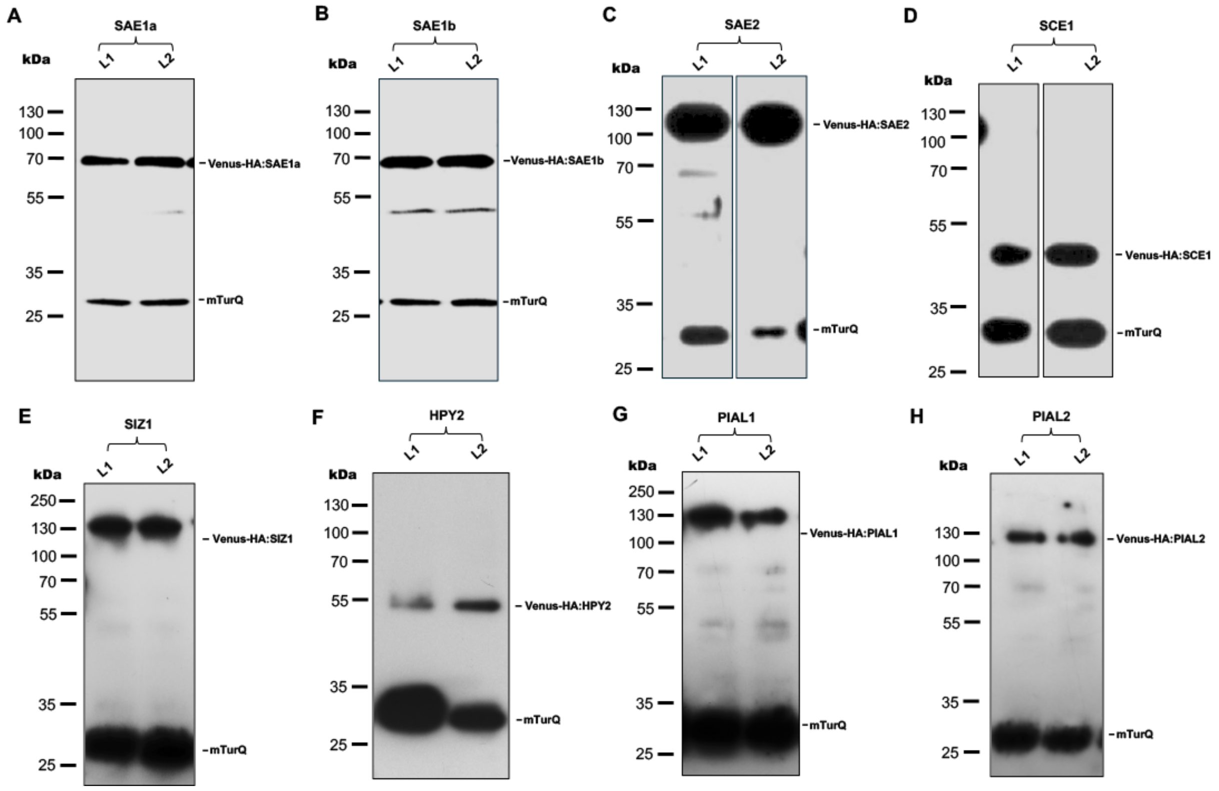

Fig. S5. Immunoblots of the E1-E4 enzyme expressing pMDS1 lines indicating efficient ribosomal skipping induced by the 2A peptides.

(A) SAE1a, (B) SAE1b, (C) SAE2, (D) SCE1, (E) SIZ1, (F) HPY2, (G) PIAL1 and (H) PIAL2. Bands corresponding to mTurquoise are labelled as mTurq.

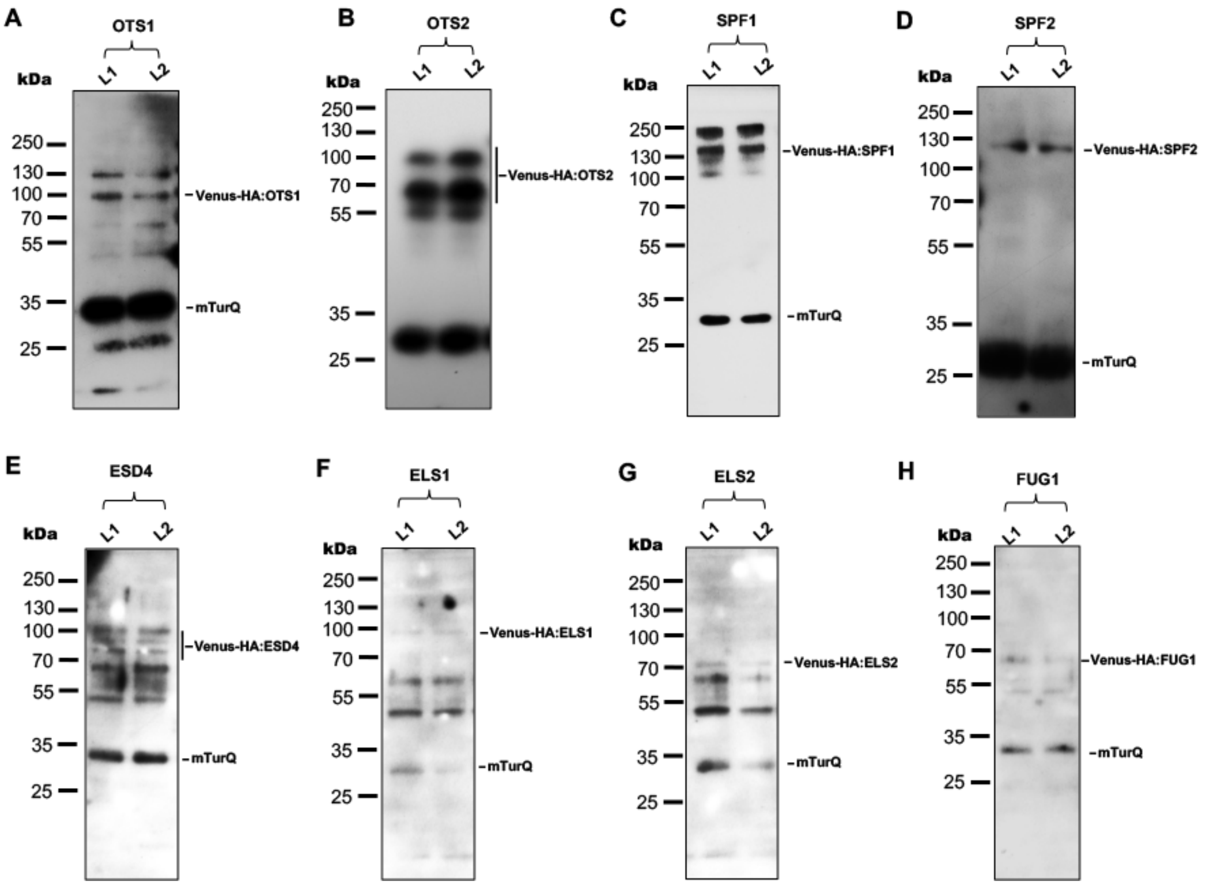

Fig. S6. Immunoblots of the ULP SUMO protease expressing pMDS1 lines indicating efficient ribosomal skipping induced by the 2A peptides.

(A) OTS1, (B) OTS2, (C) SPF1, (D) SPF2, (E) ESD4, (F) ELS1, (G) ELS2, (H) FUG1. Bands corresponding to mTurquoise are labelled as mTurq.

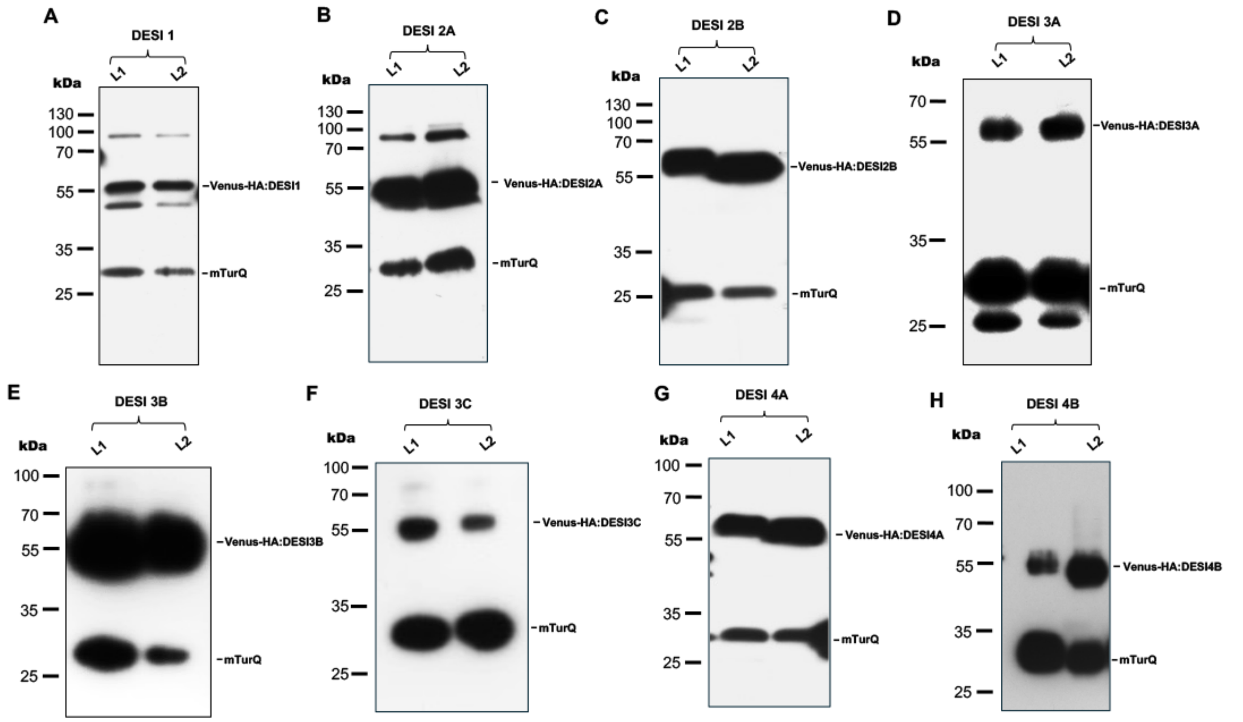

Fig. S7. Immunoblots of the DeSI SUMO protease expressing pMDS1 lines indicating efficient ribosomal skipping induced by the 2A peptides.

(A) DeSI1, (B) DeSI 2a, (C) DeSI 2b, (D) DeSI 3a, (E) DeSI 3b, (F) DeSI 3c, (G) DeSI 4a and (H) DeSI 4b. Bands corresponding to mTurquoise are labelled as mTurq.

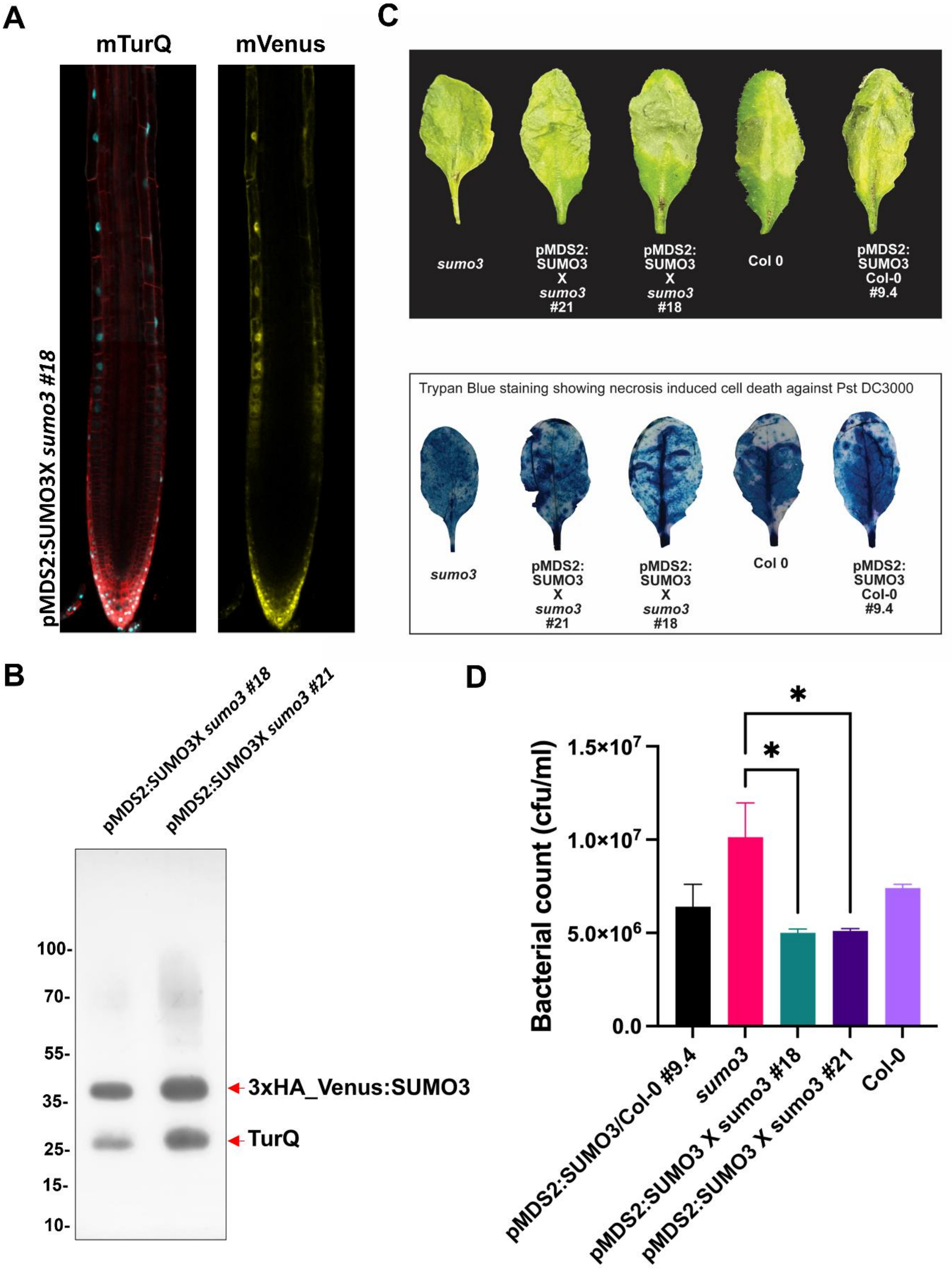

Fig. S8. **Complementation assay of *sumo3-1* mutant using pMDS2:SUMO3.**

(A) Confocal image of pMDS2:SUMO3 x *sumo3-1* showing gene expression (mTurQ) and protein level (mVenus) in epidermis. (B) Western blot analysis showing confirmation of complementation in pMDS2:SUMO3 x *sumo3-1* (Line 18 and 21). The blots were probed with α-GFP (C) Disease symptoms in leaf tissues infected with *Pseudomonas syringae pv. tomato DC3000* (upper panel), and trypan blue staining showing cell death against *Pst.* DC3000 (lower panel). (D) Bacterial count from 4-week-old Arabidopsis plants infected with virulent *Pst.* DC3000 at 3dpi. Error bars show standard error of three biological replicates. The asterisk indicates significant difference at p-value ≤ 0.05.

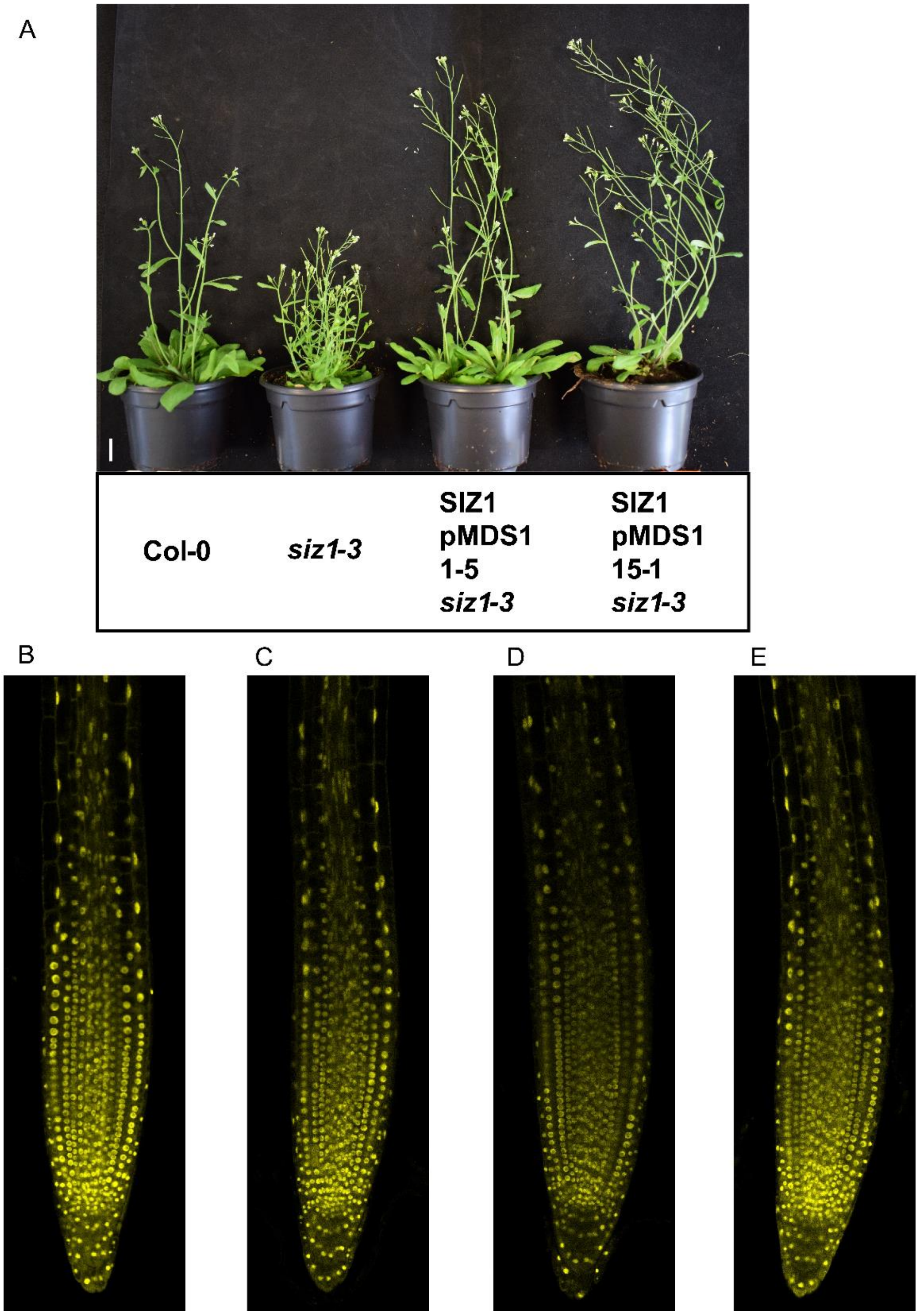

Fig. S9. Complementation assay of *siz1-3* mutant using pMDS1:SIZ1.

(A) Complementation of *siz1-3* mutant using the SIZ1 pMDS1 construct restores shoot growth. (B and C) mVenus signal of SIZ1 pMDS1 in Col-0. (D and E) mVenus signal of SIZ1 pMDS1 in *siz1-3*. Scale bars represent 1cm (A) and 100μm (B-E).

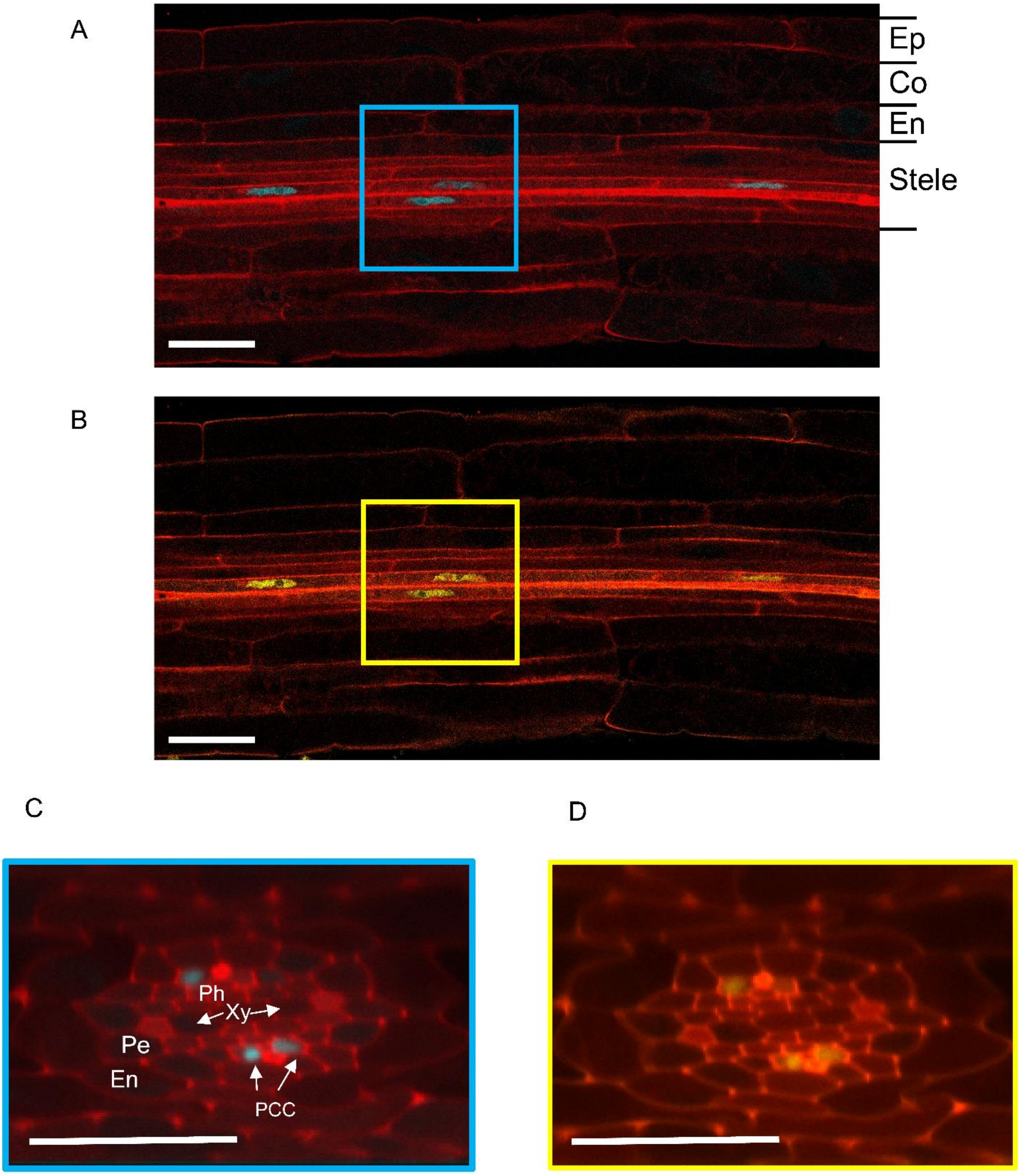

Fig. S10. SUMO1 root cross sections indicated expression and localisation in the phloem pole companion cells.

(A and B) Longitudinal sections of the localization of SUMO1 expression (A) and protein localization (B) in the root tip stele. Note the indication of the root cell type radial layout including, Epidermis (Ep), Cortex (Co), Endodermis (En) and Stele. (C and D) cross sections of localisation of SUMO1 expression (C) and protein localization (B) in the phloem pole

companion cells. Note the indication of the Endodermis (En), Pericycle (Pe), Xylem (Xy), Phloem (Ph) and Phloem Pole Companion cells (PCC). Scale bar represent 25µm.

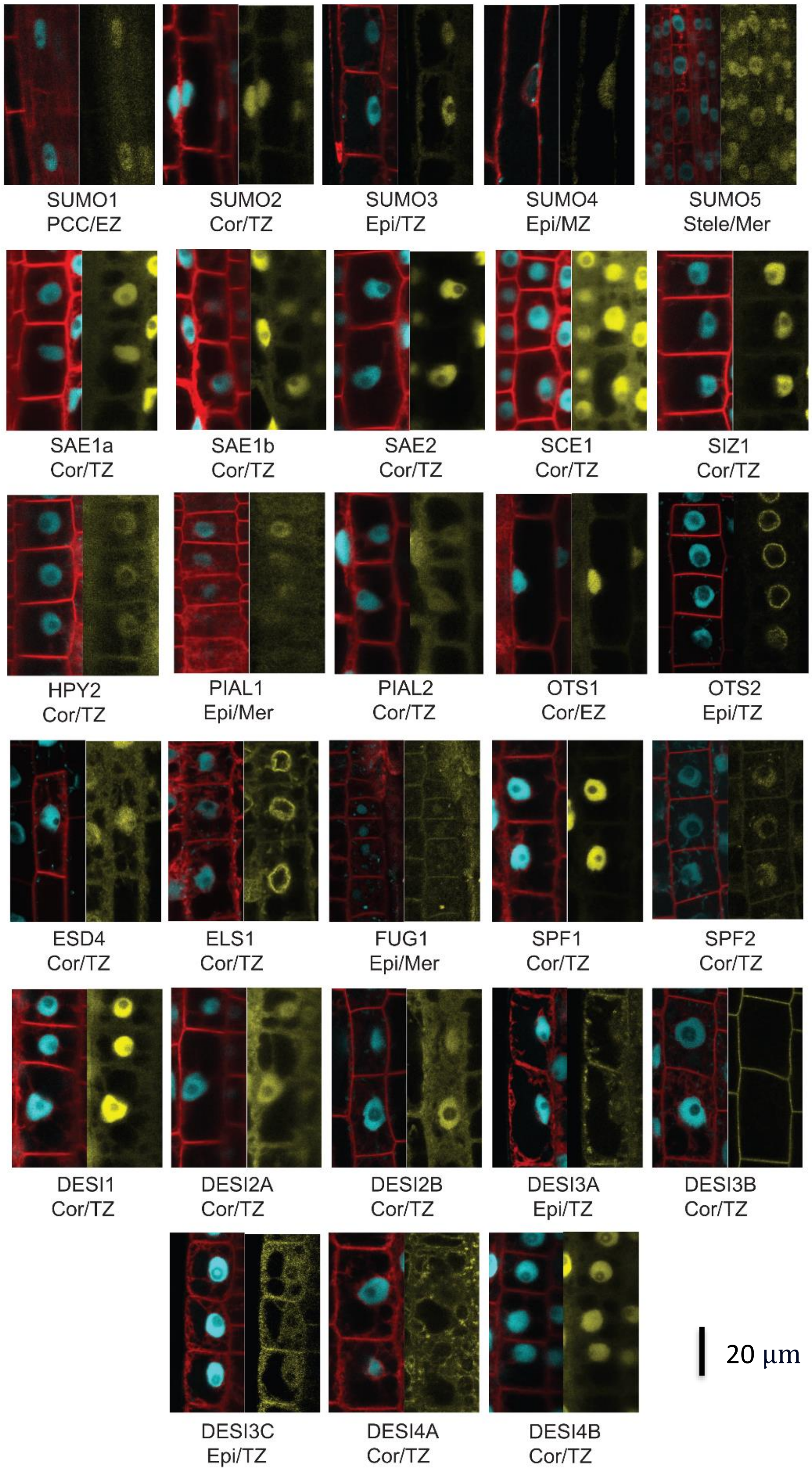

Fig. S11. **Subcellular location of all SUMO components in the root tip of A. thaliana.**

Left half of image represents mTurquoiuse (nuclear) and mCherry (membrane), while right side depicts protein localization (mVenus). Images were taken from cortex (Cor) cells in transition zone (TZ) when expressed there, in other cases the cell type/zone with the highest expression was taken. Images were taken using the Leica SP8 63x objective. Cell type abbreviations: Cor = Cortex; Epi = Epidermis; PCC = Phloem Pole Companion cells. Root zone abbreviations: Mer = Meristematic zone; TZ = Transition Zone; EZ = Elongation Zone and MZ = Maturation Zone.

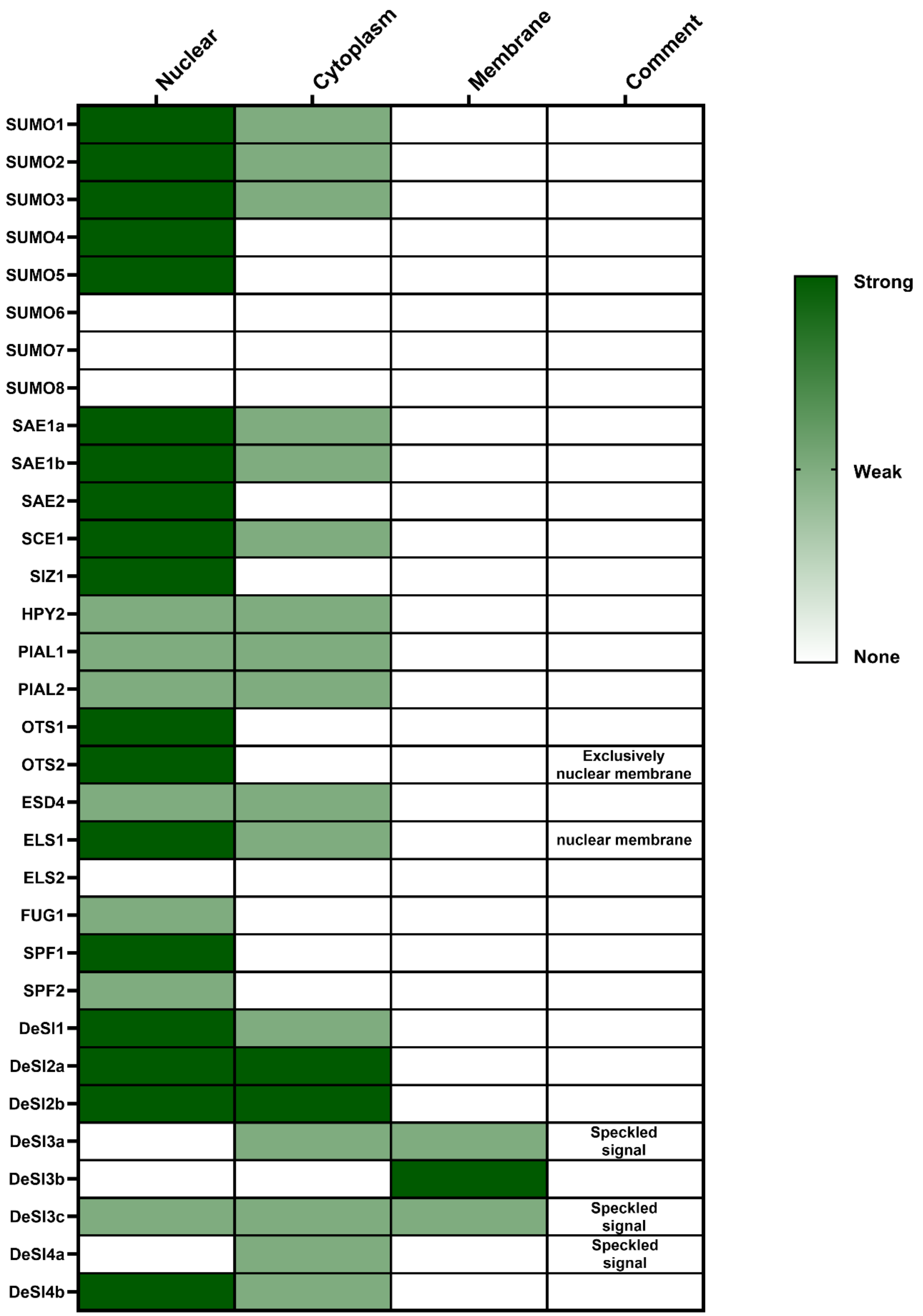

Fig. S12. **Heatmap displaying subcellular location of all SUMO components in *A. thaliana* based on imaging data in the root tip.**

The columns in the heatmap describe different subcellular locations including nuclear, cytoplasmic and membrane. The last column is used in case protein location is more specific than the previous defined groups. The colours define the strength of localisation in a certain organelle, where white defines no protein, pale green is weakly localised to this organelle and dark green is strong localisation in this organelle. For example, SUMO1 is strongly localized in the nuclear with only some expression in the cytoplasm.

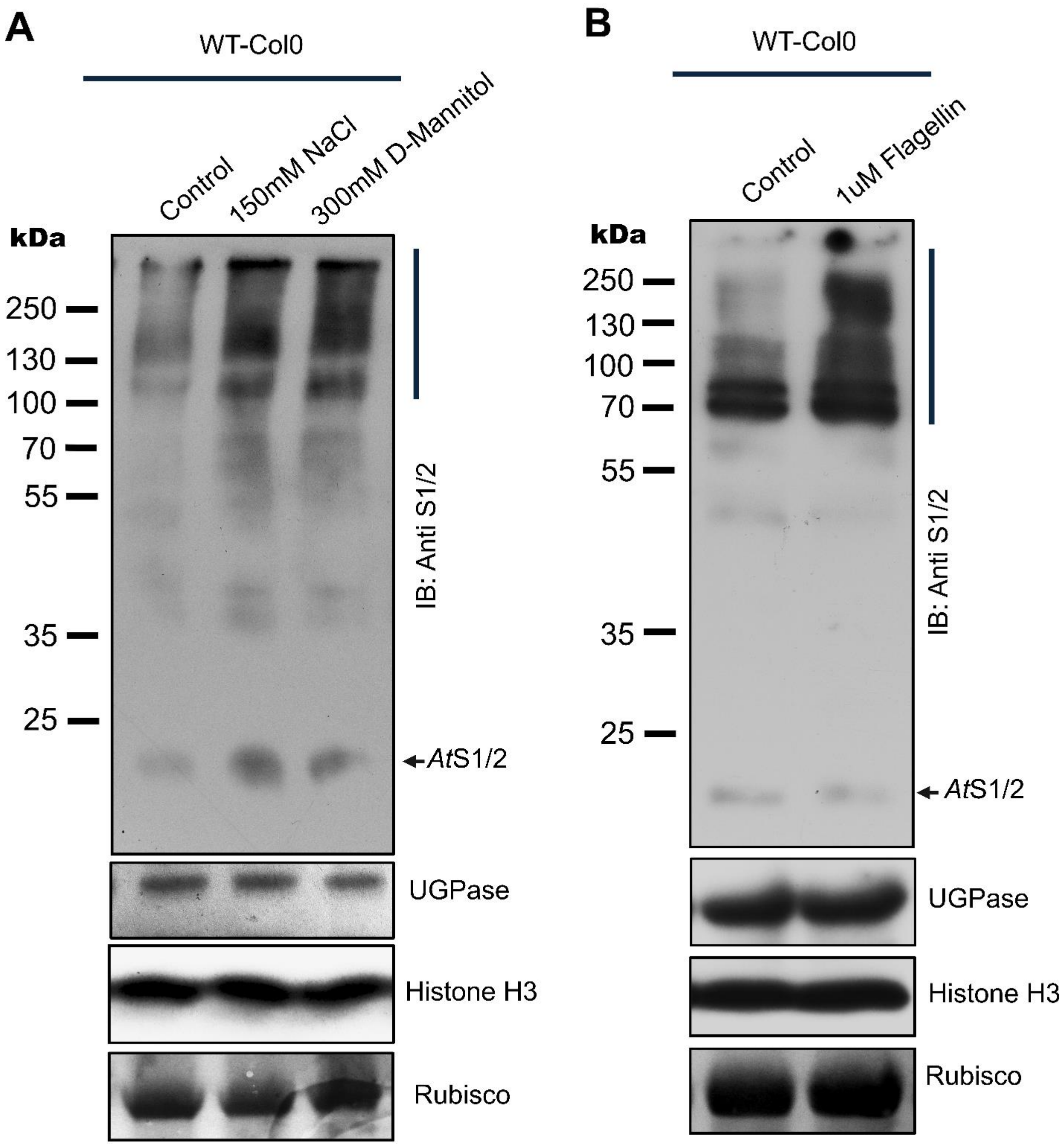

Fig. S13. SUMO1/2 conjugates accumulate with osmotic stress.

Immunoblot (IB) analysis of total protein (20 μg loaded for each lane) derived from wild-type Col-0 Arabidopsis seedlings grown for 6 days under long-day conditions and then subjected to the indicated stresses for 3 hours. Filters were probed with anti-SUMO1/2 (anti At S1/2) antibodies (J46). The vertical bar shows the laddering effect produced by the increased accumulation of SUMO1/2 conjugates. The arrowhead indicates free (nonconjugated) SUMO1/2. The bottom panels show immunostaining of UGPase and Ponceau staining of ribulose-1,5-bis-phosphate carboxylase/oxygenase (Rubisco) small subunit (RbscS), which served as loading controls. The experiment was repeated three times with similar results. (A)

Comparison of the levels of SUMO conjugate accumulation between salt and mannitol treated and control samples. (B) Comparison of the levels of SUMO conjugate accumulation between flagellin treated and control samples.

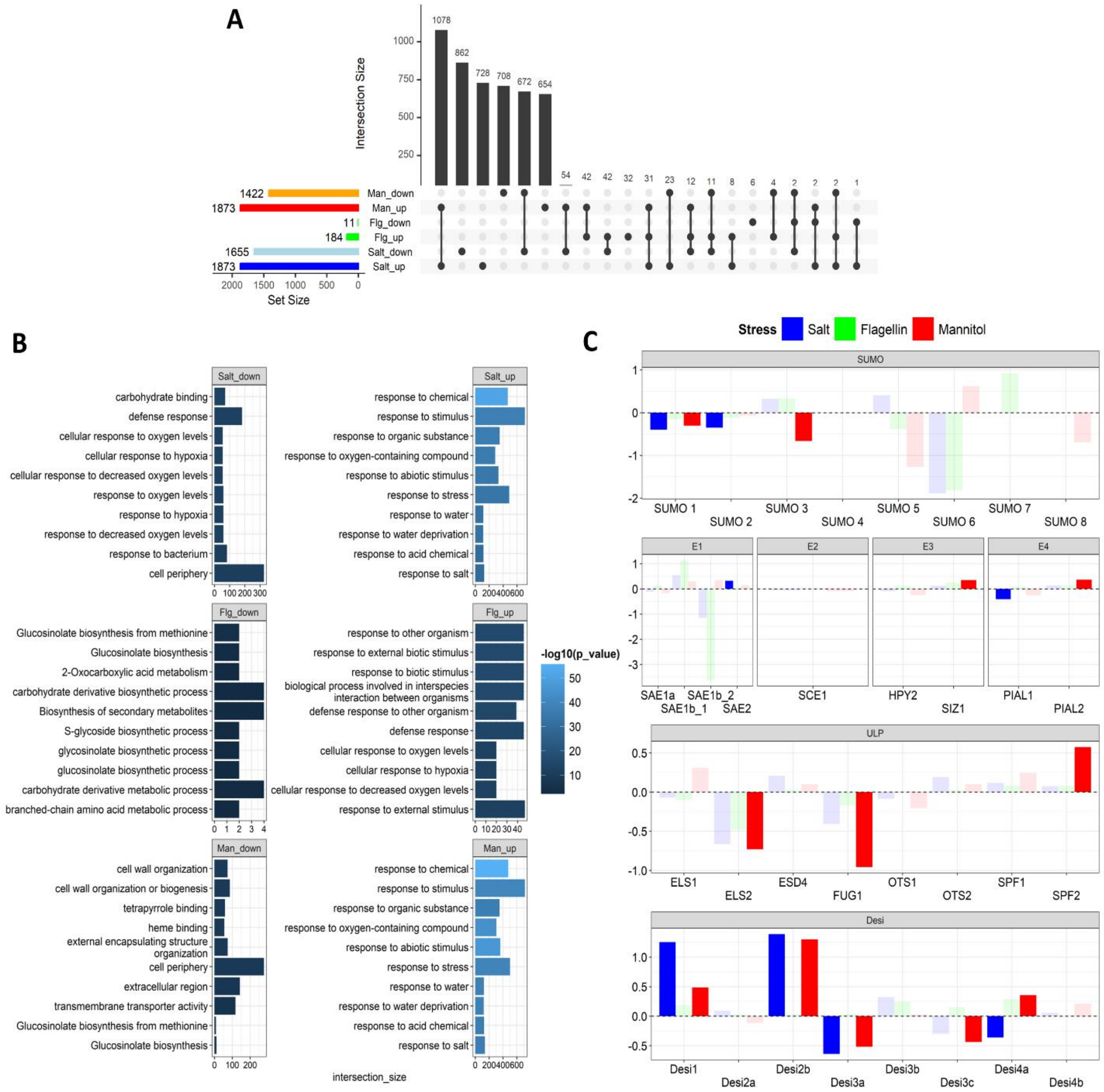

Fig. S14. Transcriptomic profiles of the SUMOylation machinery under salt, flagellin and mannitol treatments.

(A) Summary of the DEGs in response to the stresses. The number of the up/down–regulated DEGs are shown in the sidebars. The number of common DEGs between the treatments (connecting dots) is shown in the black bars. (B) Top 10 GO terms of the DEGs. The GO terms are arranged by most significant p-value (C) Transcripts expression of SUMOylation genes. The Y-axis is Log2-fold change values. Colours represent the treatments; salt (blue), flagellin (green), and mannitol (red). Solid colours are the DEG significant at p-value < 0.05.

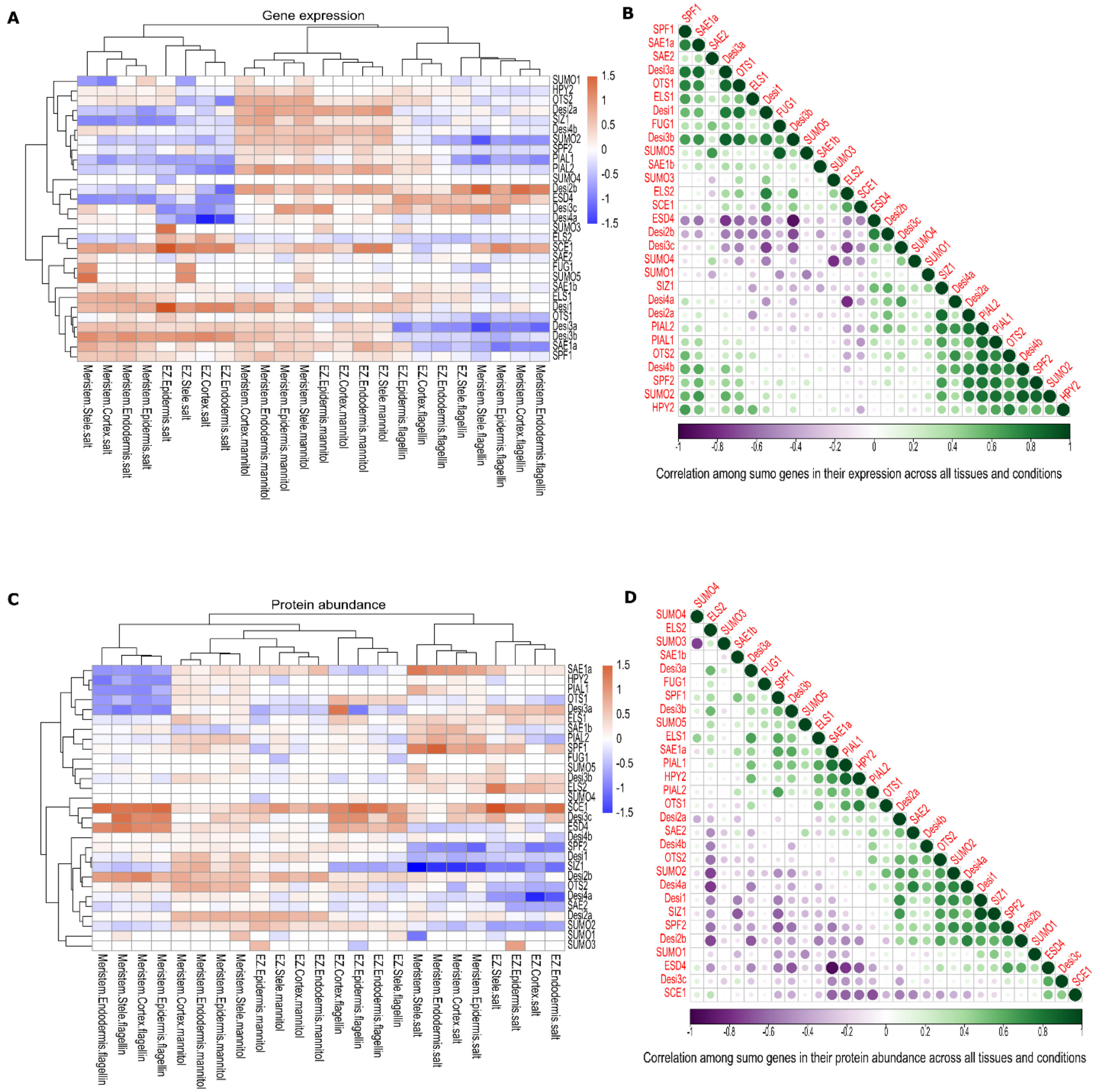

Fig. S15. Correlation analysis on the patterns of expression and protein abundance of the SUMO system genes in all 3 stresses, and across tissue types.

(A, C) The heatmaps show log2 fold change values under stress for each tissue type. The genes were clustered based on Pearson's correlation grouping those that may have similar (A) gene expression or (C) protein abundance. (B, D) The plots show correlation among sumo system genes in their (B) gene expression and (D) protein abundance, respectively. The colour and size of each dot shows the correlation value between a pair of sumo system genes when compared for all tissues and conditions at a time. The ordering of the correlation matrix was done using the angular order of the eigenvectors(AOE) parameter in corrplot R package.

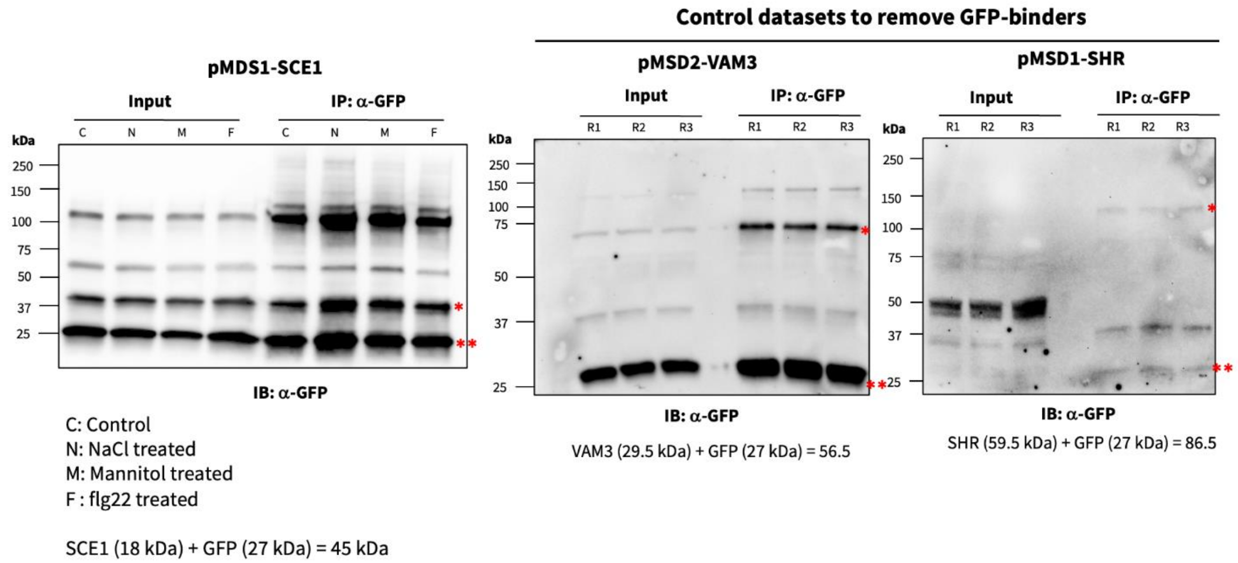

Fig. S16. Immunoblot analysis of SCE1, VAM3, and SHR following immunoaffinity enrichment using anti-GFP magnetic beads.

Single red asterisk indicates the Venus -3X HA fusion of the protein of interest (SCE1, VAM3 and SHR); while double red asterisk indicates mTurquoise band.

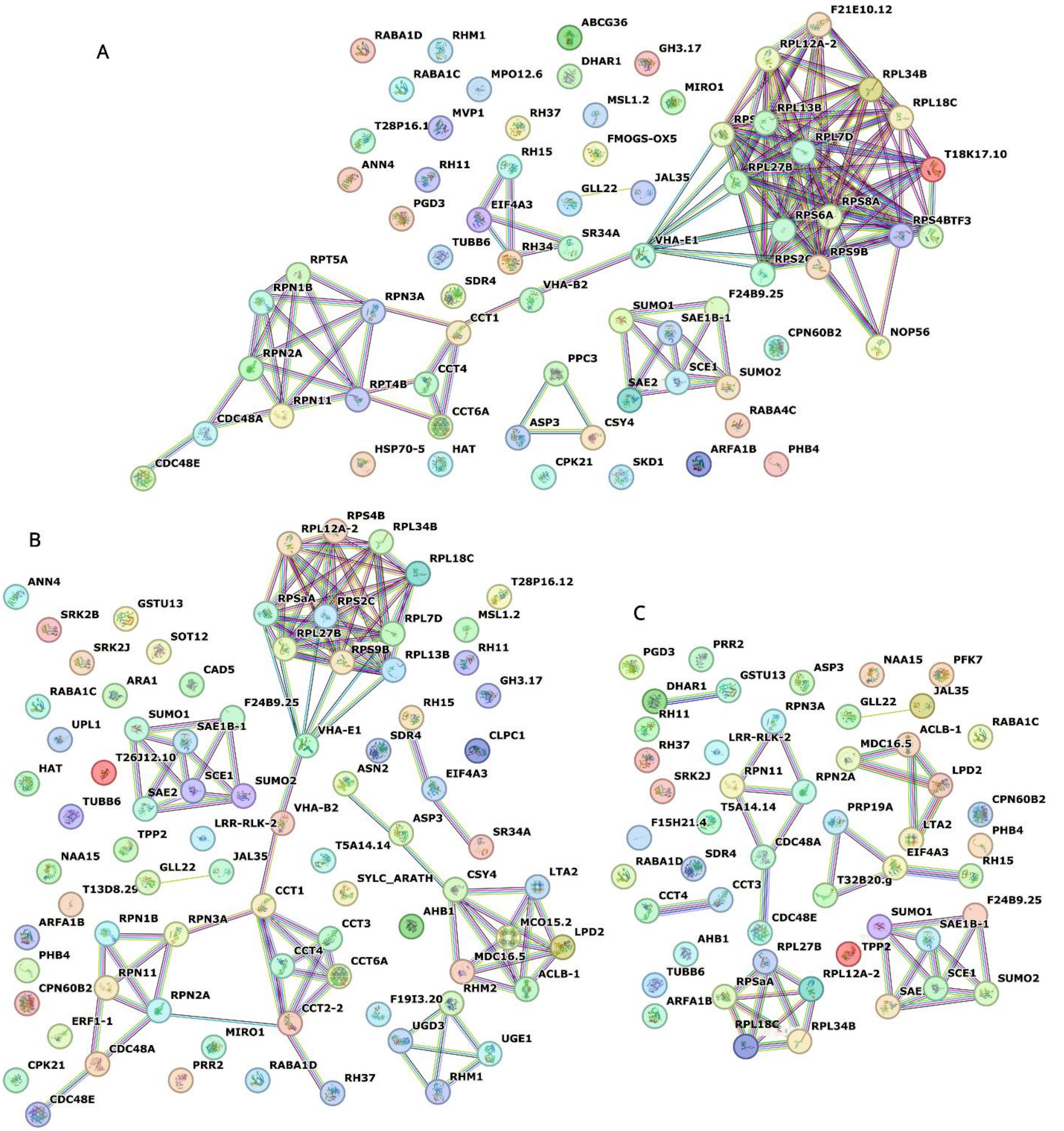

Fig. S17. **Visualization of protein-protein interaction (PPI) networks for SCE1 interactors under stress conditions [salt (A), flg22(B) and mannitol (C)] generated using the STRING database (https://string-db.org).**

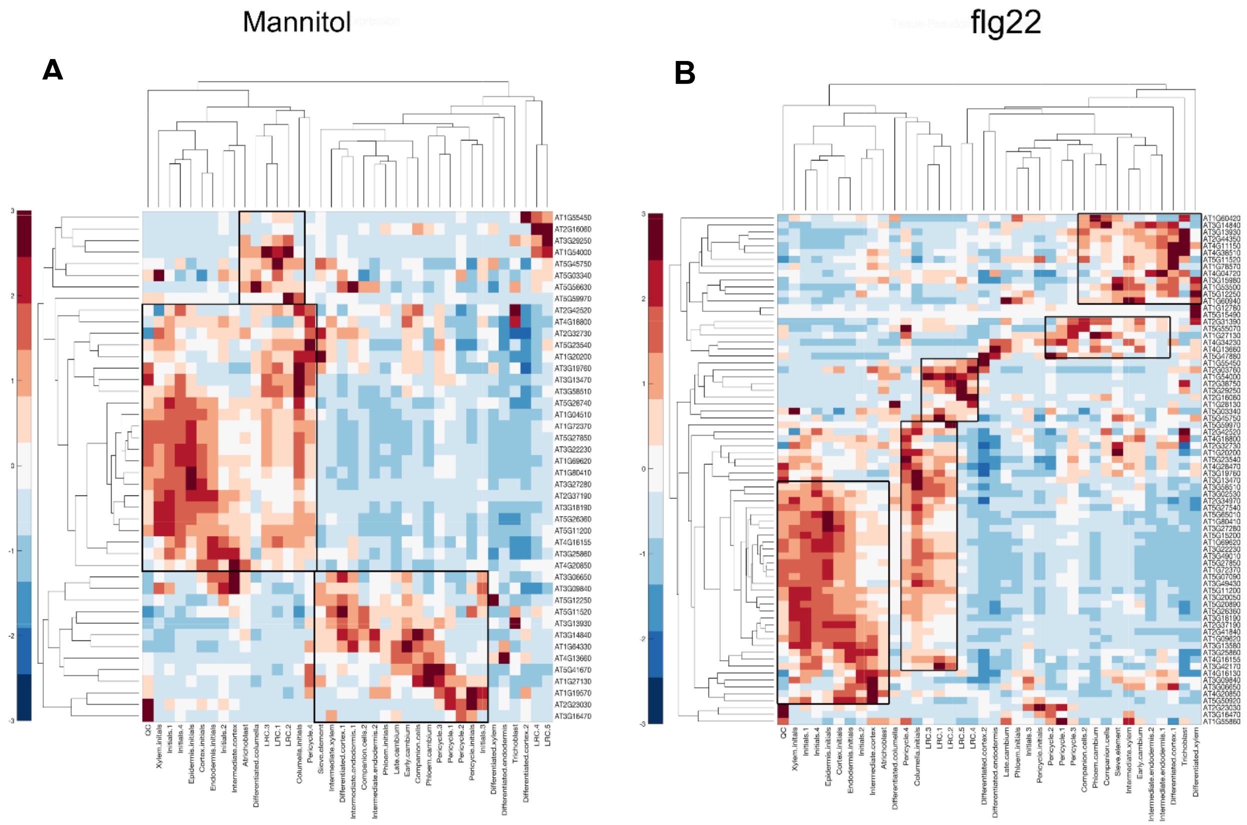

Fig. S18. Expression analysis of SCE1 interactors identified from mannitol and flg22 stress experiments across root tissues and their pseudo-time trajectories.

The heatmap displays the proportion Z-score (-3 to 3) of standardized gene expression profiles across tissue types and zones after mannitol (A) and flg22 (B) stress. The red-blue colormap represents relative expression levels, with red indicating upregulation (+1 to +3 standard deviations above the mean) and blue indicating downregulation (-1 to -3 standard deviations below the mean), with the mean represented as 0. Black boxes highlight qualitative cluster groups, and dendrograms depict hierarchical clustering of genes (rows) and zones (columns) based on expression similarity.

ID Number	Name	Primer
863	pMDS1_Venus_F	tgattacgccaagcttgggtATGGTGAGCAAGGGCGAG
864	pMDS1_Venus_R	tatgggtaaaagatgttaatGCCATCATGCTTGTACAGCTGC
868	GSG-2a-mTurq_VenusHA_F	acgttccagattacgcttccGGCTCGGGCCAGCTGTTgaattttg
79	GFP_R	CTTCTCGTTGGGGTCTTTGC
869	GFP_F_2	GCAAAGACCCCAACGAGAAG
870	N7_NLS_R	gatttcagcgtaccgacaccTTACTCTTCTTCTTGATCAGCTTCTGTGTCGTC
874	Turq_F	gttgaattttgaccttcttaAGCTTGCGGGAGACGTCG
875	Sap_Educ_F	caagcgtgaGgagcaagcaagga
876	Sap_Educ_R	tccttgcttgctcCtcacgcttg
877	Turq_R	ggctttttcggtttcgaagaAGCTTCTTCTTCCTCACCCTCGTTTC
893	3XHA_in_pMCY2_F	gcggtatcgctcttcATGgatgggcattaacatcttttaccc
867	3XHA_in_pMCY2_R	gtgatttcagcgtaccgacaCCTTAGGAAGCGTAATCTGGAACGTCATATG
880	G4S4_Venus_F	ACGTTCCAGATTACGCTTCCGGAGGTGGTGGTTCTG
881	G4S4_Venus_R	GATTTCAGCGTACCGACACCTTAGGATCATGCTTGTACAGCTCG
886	GS_2A_mTurrq_F	acgagctgtacaagcatgatTCCGGAGGTGGTGGTTCTGGTGGGGGTGGCTCctcagg
888	GS_2A_mTurrq_R	GTTCTGGTGGGGGTGGCTCctcaggTGGCTCgggccagctgttgaatttt
870	N7_pMCY2_R	gatttcagcgtaccgacaccTTACTCTTCTTCTTGATCAGCTTCTGTGTCGTC

Table S1. Primer list for generation of pMDS1 and PMDS2 vectors

Gene	Construct	Forward primer	Reverse primer
SUMO1 Promoter	pMDS2	gatcggggcgcgccctcgagactttaccataatcttcagttaaag	gtaaaagatgttaatgccatCGTGAAAGGTTCTCCGTC
SUMO1 Gene	pMDS2	gttctggtgggggtggctccATGTCTGCAAACCAGGAGGAAG	aacagctggcccgagccaccGGCCGTAGCACCACCACCGCTG
SUMO1 terminator	pMDS2	agaagagtaacacttcgtccaggtttag	aaaagctggagctccaccgcgtttaagtaatattttgtttagttgac
SUMO2 Promoter	pMDS2	gatcggggcgcgccctcgagggaataatttggctggctttgtg	gtaaaagatgttaatgcccatcttactttattatcagattatac
SUMO2 Gene	pMDS2	gttctggtgggggtggctccATGTCTGCTACTCCGGAAGAAG	aacagctggcccgagccaccAAAGCAGAAGAGCTTCAGGCCA
SUMO3 Promoter	pMDS2	gatcggggcgcgccctcgagtggggccatgtgtcattcat	gtaaaagatgttaatgcccatctttccttttatcagattttatg
SUMO3 Gene	pMDS2	gttctggtgggggtggctccATGTCTAACCCTCAAGATGACAAG	aacagctggcccgagccaccAAGCCCATTATGATCGAAAAGC
SUMO4 Promoter	pMDS2	gatcggggcgcgccctcgaggcagcacgagactcaacttt	gtaaaagatgttaatgcccattatctccactggttggatcag
SUMO4 Gene	pMDS2	gttctggtgggggtggctccATGTCAACGAAGAGCAGTAGTAT	aacagctggcccgagccaccAACTCTAAATACTGTAGAAGGACC
SUMO5 Promoter	pMDS2	gatcggggcgcgccctcgagggaagcaaggtgcatcagtt	gtaaaagatgttaatgcccattgttgttttgagaaacctt
SUMO5 Gene	pMDS2	gttctggtgggggtggctccATGGTGAGTTCCACAGACACAATC	aacagctggcccgagccaccAGGAGTGTAAGGACCGCCACCAC
SUMO6 Promoter	pMDS2	gatcggggcgcgccctcgagtggtccaataattagtgtaggct	gtaaaagatgttaatgcccattatctcaaccggtctgaacag
SUMO6 Gene	pMDS2	gttctggtgggggtggctccATGTCAACAACGAGCAGAGTTGG	aacagctggcccgagccaccAACTCTAAATTTTATAGAAGATG
SUMO7 Promoter	pMDS2	gatcggggcgcgccctcgagCGgtgcgtttaaatctcctc	gtaaaagatgttaatgcccatcgtacggagctcaacgtccctc
SUMO7 Gene	pMDS2	gttctggtgggggtggctccATGATGCAAGCATATTCCGACA	aacagctggcccgagccaccATGGCGATGGCTGAACCCTGCTA
SUMO8 Promoter	pMDS2	gatcggggcgcgccctcgagGTTTGTTCGCATGGAAGTTGC	gtaaaagatgttaatgcccatcgcttcgctttctattatg
SUMO8 Gene	pMDS2	gttctggtgggggtggctccATGTCGTCGTCTGACAAGAAAC	aacagctggcccgagccaccATGGCGATGAAAGAAGCTGAACC
SAE1a	pMDS1	gatcggggcgcgccctcgagCACAAAGGTAACCTTAGTGGCC	cagaaccaccacctccggatGAGGTAAAAGAGTCGGAAATGTCT
SAE1b	pMDS1	gatcggggcgcgccctcgagGATTGTGCTTTTTTTTCTTCTCTCAC	cagaaccaccacctccggatAGCTTGTGGGATAGGTCCTC

		T	
SAE2	pMDS1	gatcggggcgcgccctcgagGGCATTTGTTGGGTTGGAAATTAG	cagaaccaccacctccggatTCAACTCTTATCTTCTTTTTGCTCAC
SCE1	pMDS1	gatcggggcgcgccctcgagTGAGAAGATTGGAGGAGCAAAAG	cagaaccaccacctccggatACAAGAGCAGGATACTGCTTGG
SIZ1	pMDS1	gatcggggcgcgccctcgagTGAAACTGCTCCGTAGAGGAC	cagaaccaccacctccggatACTCCGGTGTCTTGTCTGATG
HPY2	pMDS1	gatcggggcgcgccctcgagGGCTGAGAGAATAAAGTCCAAGC	cagaaccaccacctccggatCCATCTTCATCCACATCTTCTGTGAAGT
PIAL1	pMDS1	gatcggggcgcgccctcgagCTAAAGGAAGCTGCTGAAATCAGA	cagaaccaccacctccggatCCCCATGTCTCAGGAGGCATTG
PIAL2	pMDS1	gatcggggcgcgccctcgagTTCATAACGACGTCTAGAATCTC	cagaaccaccacctccggatGATGTCGGTCCAGTAGGC
OTS1	pMDS1	gatcggggcgcgccctcgagAGAATGGTTGTGGATTGAAG	cagaaccaccacctccggatTCTGTCTGGTCACTGACAC
OTS2	pMDS1	gatcggggcgcgccctcgagCATTATACTTGGACGGATC	cagaaccaccacctccggatCCATCTGTTTGGTTACCC
ESD4	pMDS1	gatcggggcgcgccctcgagGGCTTTAGTCTCTCAATCTCTTG	cagaaccaccacctccggatCCATCAGCTCGTAGCCTC
ELS1	pMDS1	gatcggggcgcgccctcgagTTGTGTCCATCTTCTTTTCTACC	cagaaccaccacctccggatTCGGCTTTCAGTTGCAGAATC
ELS2	pMDS1	gatcggggcgcgccctcgagATATGAGTTACCTCTTTGGATCGGAATATTTG	cagaaccaccacctccggatCCGTCAGCTCGCAGTCTC
FUG1	pMDS1	gatcggggcgcgccctcgagTTATTATAAACCAAAAAAACTTTTTATATAAAATAAC	cagaaccaccacctccggatCCATGAATAGTTCCCAAAG
SPF1	pMDS1	gatcggggcgcgccctcgagGAGGCTTGATTGATCGGTTC	cagaaccaccacctccggatTTCTCCATCTCCTCAGCTTC
SPF2	pMDS1	gatcggggcgcgccctcgagGGAAGAGGTTGTTGTTGG	cagaaccaccacctccggatGTTTTTGGCTTGGCCATC
DeSI1	pMDS1	gatcggggcgcgccctcgagAGATGTATAGACAAGCAGAC	cagaaccaccacctccggatGCGTTTACATTGAGATGTC
DeSI2a	pMDS1	gatcggggcgcgccctcgagTGTACTTGTTAAACCATCTC	cagaaccaccacctccggatTGAAATAGTAGAACATCAGAG
DeSI2b	pMDS1	gatcggggcgcgccctcgagTGTGATCCATTATTGCTTG	cagaaccaccacctccggatTGAAACAGTAGAACATCAG
DeSI3a	pMDS1	gatcggggcgcgccctcgagCTCCATTCTTGATCTGATC	cagaaccaccacctccggatCTTTCTTTCAAGGAGCTG

DeSI3b	pMDS1	gatcggggcgcgccctcgagAAGACGGTCTCTGATTGTG	cagaaccaccacctccggatGTCTTGATGCTTACGCTTG
DeSI3c	pMDS1	gatcggggcgcgccctcgagATCATACGTTATTTACTTGTCCC	cagaaccaccacctccggatACACTCACAGTTGGTGATG
DeSI4a	pMDS1	gatcggggcgcgccctcgagCTGAAATTCCATCTGAATCGAAC	cagaaccaccacctccggatGGGATCTCCTCTCTGGTAAG
DeSI4b	pMDS1	gatcggggcgcgccctcgagGCTACGAGAACCAAGAAC	cagaaccaccacctccggatGACCGCTTCAACAAGTAC

Table S2. List of SUMO gene specific cloning primers for pMDS1 and pMDS2.

**Gene**	**Gene Identifier**	**Construct**	**Backbone**	**Bacterial host**	**Plant host**	**NASC Identifier**
SUMO1	AT4G26840	pSUMO1::mVenus-SUMO1	pMDS2	E.coli and A. tumefaciens	Col-0	2112352
SUMO2	AT5G55160	pSUMO2::mVenus-SUMO2	pMDS2	E.coli and A. tumefaciens	Col-0	2112353
SUMO3	AT5G55170	pSUMO3::mVenus-SUMO3	pMDS2	E.coli and A. tumefaciens	Col-0	2112354
SUMO4	AT5G48700	pSUMO4::mVenus-SUMO4	pMDS2	E.coli and A. tumefaciens	Col-0	2112355
SUMO5	AT2G32765	pSUMO5::mVenus-SUMO5	pMDS2	E.coli and A. tumefaciens	Col-0	2112356
SUMO6	AT5G48710	pSUMO6::mVenus-SUMO6	pMDS2	E.coli and A. tumefaciens	Col-0	2112357
SUMO7	AT5G55855	pSUMO7::mVenus-SUMO7	pMDS2	E.coli and A. tumefaciens	Col-0	2112358
SUMO8	AT5G55856	pSUMO8::mVenus-SUMO8	pMDS2	E.coli and A. tumefaciens	Col-0	2112359
SAE1a	AT4G24940	pSAE1a::SAE1a-mVenus	pMDS1	E.coli and A. tumefaciens	Col-0	2112360

SAE1b	AT5G50580/AT5G50680	pSAE1b::SAE1b-mVenus	pMDS1	E.coli and A. tumefaciens	Col-0	2112361
SAE2	AT2G21470	pSAE2::SAE2-mVenus	pMDS1	E.coli and A. tumefaciens	Col-0	2112362
SCE1	AT3G57870	pSCE1::SCE1-mVenus	pMDS1	E.coli and A. tumefaciens	Col-0	2112363
SIZ1	AT5G60410	pSIZ1::SIZ1-mVenus	pMDS1	E.coli and A. tumefaciens	Col-0	2112364
HPY2/MMS21	AT3G15150	pHPY2::HPY2-mVenus	pMDS1	E.coli and A. tumefaciens	Col-0	2112365
PIAL1	AT1G08910	pPIAL1::PIAL1-mVenus	pMDS1	E.coli and A. tumefaciens	Col-0	2112366
PIAL2	AT5G41580	pPIAL2::PIAL2-mVenus	pMDS1	E.coli and A. tumefaciens	Col-0	2112367
OTS1	AT1G60220	pOTS1::OTS1-mVenus	pMDS1	E.coli and A. tumefaciens	Col-0	2112368
OTS2	AT1G10570	pOTS2::OTS2-mVenus	pMDS1	E.coli and A. tumefaciens	Col-0	2112369
ESD4	AT4G15880	pESD4::ESD4-mVenus	pMDS1	E.coli and A. tumefaciens	Col-0	2112370

ELS1	AT3G06910	pELS1::ELS1-mVenus	pMDS1	E.coli and A. tumefaciens	Col-0	2112371
ELS2	AT4G00690	pELS2::ELS2-mVenus	pMDS1	E.coli and A. tumefaciens	Col-0	2112372
SPF1	AT1G09730	pSPF1::SPF1-mVenus	pMDS1	E.coli and A. tumefaciens	Col-0	2112373
SPF2	AT4G33620	pSPF2::SPF2-mVenus	pMDS1	E.coli and A. tumefaciens	Col-0	2112374
FUG1	AT3G48480	pFUG1::FUG1-mVenus	pMDS1	E.coli and A. tumefaciens	Col-0	2112375
DeSI1	AT3G07090	pDeSI1::DeSI1-mVenus	pMDS1	E.coli and A. tumefaciens	Col-0	2112376
DeSI2a	AT4G25660	pDeSI2a:DeSI2a-mVenus	pMDS1	E.coli and A. tumefaciens	Col-0	2112377
DeSI2b	AT4G25680	pDeSI2b::DeSI2b-mVenus	pMDS1	E.coli and A. tumefaciens	Col-0	2112378
DeSI3a	AT1G47740	pDeSI3a::DeSI3a-mVenus	pMDS1	E.coli and A. tumefaciens	Col-0	2112379
DeSI3b	AT2G25190	pDeSI3b::DeSI3b-mVenus	pMDS1	E.coli and A. tumefaciens	Col-0	2112380

DeSI3c	AT5G25170	pDeSI3c::DeSI3c-mVenus	pMDS1	E.coli and A. tumefaciens	Col-0	2112381
DeSI4a	AT4G17486	pDeSI4a::DeSI4a-mVenus	pMDS1	E.coli and A. tumefaciens	Col-0	2112382
DeSI4b	AT5G47310	pDeSI4b::DeSI4b-mVenus	pMDS1	E.coli and A. tumefaciens	Col-0	2112383

Table S3. List of all constructs and plant lines made in this paper with NASC code.

**Relative Level**	**Level of fluorescence**
Not detectable	Below detection threshold
Very low	Barely visible at 26% laser, pixel values (pv) below 50
Low	Visible at 26% laser, but barely visible at 14L (pv < 50)
Medium	Visible but not overexposed at 14% laser (pv between 50 and 200)
High	Overexposed at 14% laser, normal level at 2% laser (pv between 50 and 200)
Very high	Bordering on overexposure or overexposed at 2% laser (pv >200)

Table S4. Relative fluorescence was scored in 6 different bins from not detectable to very high level of fluorescence.

Name	Forward primer	Reverse primer
pMDS1_N7	gaGgagcaagcaaggaaagc	gatttcagcgtaccgacaCC
PP2A	TAACGTGGCCAAAATGATGC	GTTCTCCACAACCGCTTGGT
ACTIN2	CCGCTCTTTCTTTCCAAGC	CCGGTACCATTGTCACACAC
SUMO1	CGTCCAGGTTTAGGGCTTGT	AGGATCCGATACCAAACGAACA
SUMO2	GGCGGTTATTTTAATGGTTTTCCA	CCAAAATCCATAAACAAGCCCA
SUMO3	TGGCTGGCTTTGTGATATTTGA	TGATGAGTTGTTTATTCACGGTTGT
SUMO4	TGAGTGTTTCTGGTGTGAATGT	ACAAGCATAATACTCGGCCCT
SUMO5	TTGTTCGCGGGATAAACAGA	ACGCTTTCTCGATATTCCGGT
SUMO6	GGAAAGGGAAGCAACAAAAGGA	CCTTCTTCGCCTACATGCCT
SUMO7	AGTCATTAGTGCGTTTACAAAAAGA	CAACTTCCATGCGAACAAACA
SUMO8	CGTTAGAACGACTCACCAAAAA	CAATGCCCATCTATTCTCATATCTC
SAE1a	GCACAGGAGGTGATCAAAGCAG	aactaaatcaaggcccactggttg
SAE1b	GAGGGATTCTAGGACAGGAGGTG	agagcagcgtttcacagcac
SAE2	GGAGGTTGAAAATCCCATGATGGT	cctgagctcctcatccaaaggatc
SCE1	TCTGTCAGGATCCAGTTGAGTACAAG	ccaaaatgaggagttttcaatcaccca
SIZ1	TGGATACTACAACAGAGACTGCGTC	ccggttcaattcttgcctgca
HPY2	TTGTCCTGTAGCAGGGTGCC	cagcgcttaaaacccggtcc
PIAL1	GGGTATTCAGGGTCGTTTTGGTCA	atacgctacatcaagaacttagcccc
PIAL2	ACAACGGCTGCCTATAGAACCG	CTCTGTTCATGGCGTGGGAAAG
OTS1	GAAGAGGCGCCTCAAAGG	TGAACTCTGTTAGAGCGGTGA
OTS2	GGCTCCTCAAAGGCTGACAT	GAGTGACCAAACTTGACACTGTTC
ESD4	CACATGCCATACTTCCGACTCAG	AGCCGAGTGCTTCACTGC
ELS1	ACCGGAGTGTTTACAAGGGT	CTTGCTTGCTTCCCGAGAGT

ELS2	AGATACTGAGACTGCGAGCTG	CATTCTCCACAGCCCAAGC
FUG1	GAGCAGGACTGCTGACGTAT	AGCAAGGCAAGACAAAACA CTC
SPF1	CTTTGGATGCGTTGGCGTTG	GGTGGGGCTTTTCCTCAGAT
SPF2	TGAGAGGAGCTCAGACGACA	CGCATTAGATTGGCGATATT CATC
DeSI1	TAGTGCAATAACAGAGAGATG	CTGCTCTTTTCCTCTTTTATG
DeSI2a	CAACTGTACTGATCCACCC	CTTGCTTGGTCTTTGCCAG
DeSI2b	GCATTCGGTTAGCTTATATC	GATTTGACAGTAACAAGCGA G
DeSI3a	GGTCTCTCATTCGTTTCGTTAG	CAGCGTTGATGCAATACATG A
DeSI3b	GACAAGAACAGAGGAACATG	GTACATACTCATGACCTCTCT
DeSI3c	CGCAATTTGATTCGGTAGTA	GGACTTGACTTCACTTTCAC
DeSI4a	CGACAATGTACTTCCTCCTGA	TACTGCCAAAGGGTTGTCTG A
DeSI4b	TGGGTCCATATATTGAGTATTCTTT GG	GAACTCACATTGGGAGAAGA AG

Table S5. Primer details used for Real Time quantitative PCR.

Data S1 Excel spreadsheet containing list of SCE1 interactors after 3hrs of salt, mannitol and flagellin stress and a list of SUMO1 targets among these interactors.
